# NMR Metabolite Profiling in the Quality and Authentication Assessment of Greek Honey—Exploitation of STOCSY for Markers Identification

**DOI:** 10.3390/foods11182853

**Published:** 2022-09-15

**Authors:** Gabriela Belén Lemus Ringele, Stavros Beteinakis, Anastasia Papachristodoulou, Evangelos Axiotis, Emmanuel Mikros, Maria Halabalaki

**Affiliations:** 1 Division of Pharmacognosy and Natural Products Chemistry, Department of Pharmacy, National and Kapodistrian University of Athens, Panepistimiopolis, Zografou, 15771 Athens, Greece; 2 Natural Products Research Center “NatProAegean”, Gera, 81106 Lesvos, Greece; 3 Division of Pharmaceutical Chemistry, Department of Pharmacy, National and Kapodistrian University of Athens, Panepistimiopolis, Zografou, 15771 Athens, Greece

**Keywords:** Greek honey, STOCSY, NMR profiling, botanical origin, geographical origin, biomarkers

## Abstract

Honey is a natural, healthy commodity and is probably among the most complex foods produced by nature. It is the oldest recorded and certainly the only natural sweetener that can be used by humans without any further processing. Nowadays, the increase in honey’s value, along with its growing list of healthy attributes, has made the present raw material a prime target for adulteration. In the current study, NMR-based metabolite profiling in combination with chemometrics was applied in the quality control of Greek honeys from northeastern Aegean islands. Moreover, statistical total correlation spectroscopy (STOCSY) was employed for the first time as a dereplication and structural elucidation tool in the honey biomarker identification process. A total of 10 compounds were successfully identified in honey total extracts via ^1^H NMR spectroscopy. Compounds such as 5-(hydroxymethyl)furfural, methyl syringate, a mono-substituted glycerol derivative and 3-hydroxy-4-phenyl-2-butanone, among others, were identified as potential biomarkers related to the botanical and geographical origin of the samples. High-Resolution Mass Spectrometry (HRMS) was used as an additional verification tool on the identified compounds.

## 1. Introduction

Honey is a natural product characterized by its sweet taste and its high viscosity. It is one of the most complex foods produced by nature [1]. Its chemical composition is related to different factors, such as climate, nectar flow, production method and storage process, but it is also strongly related to its botanical and geographical origin [2]. The significance of honey has increased because of the nutrients and therapeutic properties it demonstrates. Thus, the mentioned factors have highlighted honey as a high-demand and high-value product, thus making it a prime target for economically motivated adulteration, leading to an increase in fraudulent cases.

In recent years, different analytical methodologies have been developed to guarantee the authenticity of this food commodity, since traditional methods, such as melissopalynological techniques (e.g., pollen analysis) and the determination of physicochemical characteristics (e.g., conductivity, pH, acidity, lightness, color, rheological properties), are time-consuming and trait specific. Different chromatographic, vibration spectroscopic and spectrometric methods in combination with chemometric tools have been adopted to determine the chemical composition of honey samples, to quantify several major compounds and, more importantly, to detect adulteration cases in commercial products. These techniques are extensively analyzed in the literature [3,4,5]. The developed methods are based on the statistical analysis of physicochemical data or on the determination of different chemical compounds that stand out as biomarkers related to specific descriptive parameters. Among all the techniques applied in food quality assessment, NMR spectroscopy is a versatile method that can provide great amounts of comprehensive information about the sample under study. It is a fast, reproducible, non-destructive technique, which can detect a wide range of chemical compounds in a single run. Therefore, NMR as an analytical platform has been increasingly applied for authentication assessment purposes in recent years.

On the other hand, statistical analysis methods have been developed to extract information from the data obtained with different analytical techniques. Nevertheless, the discovery of quality markers (biomarkers) remains a challenging step due to the intricate physicochemical properties of honey. Moreover, inherent problems in foodomics, such as sample complexity and variability, the scarcity of databases and the reliable structure identification of biomarkers, further undermine this process. To that end, statistical total correlation spectroscopy (STOCSY) is a statistical tool introduced in 2005 for NMR spectra. STOCSY takes advantage of the multi-co-linearity of the intensity variables in a dataset to generate a pseudo-two-dimensional NMR spectrum that displays the correlation among the intensities of various peaks across the whole sample [6]. Until today, it has been employed mainly in human or animal biofluids (serum, plasma, urine, etc.), while there are very few applications in the field of natural products. Recently, it has been successfully applied for the first time in the analysis of edible olives, allowing several biomarkers to be accurately identified [7].

Regarding honey, several studies have been carried out concerning its authenticity assessment. However, only one study has previously been performed about the quality and authenticity assessment of Greek honey by means of NMR spectroscopy, and it was only limited to the analysis of the sugar part of the material and the determination of its chemical composition [8]. Therefore, the aim of this study was to investigate the application of NMR-based metabolite profiling in combination with chemometrics in the quality control of Greek honeys originating from northeastern Aegean islands by studying their entire metabolic profile after the elimination of sugars. Additionally, the premiere evaluation and application of STOCSY as a dereplication and structural elucidation tool in the biomarker identification process of honey was studied. To our knowledge, this is the first time in which NMR-based metabolite profiling is applied in non-sugar fractions in Greek honey.

## 2. Materials and Methods

### 2.1. Chemicals and Materials

Distilled water (d-H_2_O) was used for the extraction of honey samples. Methanol (CH_3_OH; analytical grade; Fisher Scientific, Loughborough, UK), ethyl acetate (EtOAc; analytical grade; Macron Fine Chemicals™ Leicestershire, England), hydrochloric acid (HCl; ACS Reagent; 37%, Honeywell Fluka™, Wien, Austria), n-butanol (n-BuOH; RPE analytical grade; Carlo Erba reagents S.A.S, Cornaredo MI, Italy), chloroform (CHCl_3_; HPLC grade; Fisher Scientific, Loughborough, UK) and dichloromethane (DCM; HPLC grade; Carlo Erba reagents S.A.S, Val-de-Reuil, France) were also employed during the development and the optimization process of the extraction protocol. XAD-type adsorbent resins (XAD-4; XAD-7HP; Amberlite; 20–60 mesh) were obtained from Merck SA (Athens, Greece). Deuterated chloroform (chloroform-*d*; 99.8% D) used for NMR analysis was acquired from Euriso-top GmbH (Saint-Aubin, France), while hexamethyldisiloxane (HMDSO; NMR grade, ≥99.5%), used as internal standard (IS), was acquired from Sigma-Aldrich Corporation (St. Louis, MO, USA).

### 2.2. Collection of Samples

Honey samples of different botanical and geographical origins were collected and provided by producers from the islands of the northeastern Aegean region of Greece, such as Chios, Lesvos, Psara, Ikaria, etc. In the present study, 76 honey samples were collected and analyzed (Table S1). Samples were stored under dark conditions at 4 °C pending analysis.

### 2.3. Sample Preparation

Honey is a demanding raw material, and its handling is a really challenging task, mainly due to its highly viscous nature. The presence of sugars in this food commodity is dominant, while non-sugar compounds occupy only a small percentage of the total matrix [9]. Thus, the first step was to produce a non-sugar-enriched extract, while attempting to remove most of the sugar content. To obtain the optimal enriched extract, several different approaches were employed.

The samples underwent different treatments using XAD-type adsorbent resins to finally achieve the highest yields of the non-sugar-enriched extracts and the highest metabolic coverage. Briefly, the optimization process of the extraction protocol is described below:

(a). First, 10 g of honey was dissolved in 100 mL of d-H_2_O, followed by the addition of 20 g of Amberlite XAD-4 adsorbent resin. The sample was stirred for 3 h. The mixture was then filtered and washed for three times with 200 mL of d-H_2_O. The resin was then transferred to a large glass container, and 100 mL of CH_3_OH was added. Non-sugar compounds were extracted with the aid of an ultrasonic bath for 20 min. Then, the resin was filtered and washed with 100 mL of CH_3_OH. The last step was repeated in triplicate. Eventually, the methanolic phases were combined and evaporated to dryness, as was performed with the aqueous phase;
(b). The procedure was repeated, but in this case, honey was dissolved in acidified d-H_2_O with pH = 2 adding concentrated HCl;
(c). A total of 6 g of honey was dissolved in 30 mL of acidified water (pH = 2) and stirred for 1 h. A total of 9 g of resin was in turn added, and the stirring continued for another hour. The next steps were performed as previously described. In the end, the methanol extract was subjected to liquid–liquid extraction (LLE) using EtOAc as organic solvent (3 × 5 mL).

The procedure with all steps was repeated using another type of adsorbent resin, Amberlite XAD-7.

Furthermore, honey samples were directly subjected to LLE using different biphasic systems:

(a). Firstly, 6 g of honey were dissolved in 15 mL of d-H_2_O and extracted with EtOAc (3 × 15 mL). Organic phases were mixed and evaporated to dryness;
(b). Another trial involved successive extraction using 15 mL of n-BuOH and EtOAc;
(c). Regarding the third treatment, extraction was performed as described by Schievano et al. [10] with the appropriate modifications. A total of 6 g of honey was weighed in a 50 mL centrifuge tube and dissolved in 15 mL of d-H_2_O. A volume of 15 mL of CHCl_3_ was added, and the mixture was shaken for 10 min in a vortex agitator. The biphasic mixture was then centrifuged at 4000 rpm (1968× *g*) for 15 min at room temperature. The organic phase was collected and evaporated to dryness. The preceding LLE was repeated using smaller quantities of honey samples—0.2 and 0.5 g of raw material with 1.6 and 4 mL of biphasic systems, respectively;
(d). A fourth trial was carried out, as described in (c) with the sole alteration being that the tube was allowed to stand for 20 h before and after centrifugation;
(e). Finally, LLE was carried out, as described in (c), using various solvent systems with DCM, EtOAc and CH_3_OH in different ratios (DCM 100%, EtOAc/DCM 90:10 *v/v*, EtOAc/DCM 80:20 *v/v*, DCM/CH_3_OH 90:10 *v/v*, EtOAc 100%).

#### Optimized Extraction Protocol

Following the optimization process, the treatment with XAD-type adsorbent resins and LLE led to the following final extraction protocol: A total of 6 g of honey was weighed in a 50 mL centrifuge tube and dissolved in 15 mL of d-H_2_O. A volume of 15 mL of organic system EtOAc/DCM (80/20 *v/v*) was then added, and the mixture was shaken for 10 min in a vortex agitator. The mixture was centrifuged at 4000 rpm (1968× *g*) for 15 min at room temperature. The organic phase was collected and evaporated to dryness. The procedure was repeated twice for each sample. After the step of extraction, samples were stored in a freezer at −20 °C pending analysis.

### 2.4. NMR Analysis

#### 2.4.1. Preparation of NMR Samples

All samples were dissolved in 650 μL of chloroform-*d*, and 600 μL was finally transferred in NMR tubes (D600-5-7) with polytetrafluoroethylene (PTFE) caps, which were obtained from Deutero GmbH (Kastellaun, Germany). HMDSO at a concentration of 0.02% *v/v* was used as an internal standard (IS) and a line–shape indicator to monitor shimming performance during acquisition.

#### 2.4.2. NMR Experimental Parameters

A Bruker AVANCE III 600 NMR spectrometer, equipped with a z-gradient inverse detection 5 mm probe (proton frequency of 600.113 MHz, B0 = 14.1 T) was employed for the acquisition of ^1^H-NMR spectra. The temperature was kept stable throughout the experiment at 305 K. Experiments were conducted with the IconNMR suite by Bruker and the help of a 60-place sample changer (B-ACS-60). Parameters for acquisition were as follows: number of scans, 64; π/2 pulse, ~8 us, time domain (TD), 64 k data points; acquisition time, 3.9 s; relaxation delay, 2 s; and spectral width, 8417.509 Hz. An exponential multiplication window function with a line-broadening factor (lb) of 0.3 Hz was applied on the free induction decay (FID) to obtain the final spectra. Manual phase- and baseline-correction was performed with Bruker TopSpin software (version 4.0.6). Chemical shifts were reported with respect to HMDSO at 0 ppm.

### 2.5. Computational Processing and Multivariate Analysis

NMR raw data were inserted into the MATLAB (version R2018a) suite using *in-house* routines for further spectral processing. Firstly, spectra were aligned using the icoshift tool (Version 3.0). Then, NMR spectra (spectral width from −0.2 to 12 ppm) were segmented into 1221 equal-sized bins using a bin size of 0.01 ppm for the multivariate analysis (MVA). Data were normalized using the IS (reference region between 0.12 and −0.12 ppm). Prior to the MVA, bins that contained solvent signal (chloroform-*d*), spectral regions with no significance (baseline noise) and the IS signal were removed to avoid any interference with and contribution to the statistical models. Moreover, NMR spectra were segmented into 12201 equal-sized bins using a bin size of 0.001 ppm for the statistical total correlation spectroscopy (STOCSY) experiments to achieve enhanced resolution.

Data with a 0.01 ppm bin size were imported into SIMCA v. 14.1 software (Sartorius, Göttingen, Germany), where they were subjected to MVA and specifically to a principal component analysis (PCA), a partial least squares discriminant analysis (PLS-DA) and an orthogonal projections to latent structures discriminant analysis (OPLS-DA). Prior to the analyses, data were mean-centered and scaled using Pareto scaling along with log-10 transformation. Outliers were detected and removed in all constructed models with Hotelling’s T2 and Distance to the Model in the X- and Y-block (DModX and DModY). To check the validity of the generated models, permutation tests were conducted using 200 random changes. Data with a 0.001 ppm bin size were used for the 1D STOCSY analyses with an *in-house* routine in the MATLAB suite. As mentioned above, regarding NMR spectroscopy, STOCSY is a technique in which a pseudo-NMR spectrum is generated to identify the correlation among different spectral features [6]. This technique is able to correlate and identify signals from the same compound according to the variance in its concentration levels within the different spectra. A threshold of 0.75 for the correlation coefficient was set on the whole dataset and kept at the same level for all the generated pseudo-spectra for comparison reasons of the different correlations between the different peaks. Therefore, the two-step approach used OPLS-DA to extract NMR chemical shifts values that were then cross-combined with the STOCSY statistical analysis, in order to strengthen the validity of the identification process of the compounds, which are responsible for any observed metabolic alteration. Additionally, the assigned metabolites were also subjected to a univariate analysis using GraphPad Prism v6.01 software (GraphPad Software, Inc., San Diego, CA, USA) for the visualization of the variance in the metabolite levels. The statistical significance was assessed with a one-way analysis of variance (ANOVA) and based on Tukey’s correction. A *p*-value ≤0.05 was considered as statistically significant for the dataset.

## 3. Results and Discussion

### 3.1. Metabolite Profiling of Honey Samples via ^1^H NMR Spectroscopy

#### 3.1.1. Collection of Samples

Initially, the collection of honey samples had to be ensured. In these first steps of mapping Greek honeys, the region of our focus was placed in the northeastern part of the Aegean, Greece, on which no study has been conducted to this day to our knowledge. This specific region is characterized by numerous isolated islands, most of which form the borders between Greece and Turkey, presenting unique biodiversity and giving additional value to the present study. More specifically, honey samples were collected from the islands of Agios Efstratios, Ikaria, Lesvos, Samos, Lemnos, Fournoi Korseon, Chios and Psara, covering different botanical origins, such as plant, spring honey/heather, chestnut/blossom and thyme. In more detail, plant and blossom honeys correspond to a mixture of honeydew and flower nectar origins, respectively, while spring honey mainly refers to the collection period of the respective samples, with no further information regarding its botanical origin being provided by the producers. Table 1 summarizes all the details concerning the samples.

#### 3.1.2. Sample Preparation

After the collection of the honey samples, the next step of our study was to optimize the extraction protocol. Two main fractions were produced from this procedure, the sugar fraction and the non-sugar one, with the latter being selected for further investigation, as it was enriched with secondary metabolites of interest. It is worth mentioning once more that there is only one study using NMR spectroscopy for authentication purposes in Greek honey; however, it focuses on the study of sugars via ^13^C NMR experiments directly on aqueous dissolved honey samples [8].

Initially, XAD-type adsorbent resins were considered. XAD-2 has previously been widely used since it allows interfering compounds to be effectively eliminated. In this case, XAD-2 was substituted by XAD-4, which has been proposed as a more efficient method for the extraction of polar compounds due to its higher porosity and surface area. In addition, the use of acidified water is usually recommended, since it allows a recovery of flavonoid aglycones in a percentage higher than 95% to be obtained [11].

However, no differences were observed in our case with the use of acidified water, and both trials led to very low yields. It is important to note that the majority of the recovered compounds consisted of sugars, as their elimination was not sufficient. A further clean-up step was deemed necessary, leading to the performance of further trials by combining different extraction procedures. The extraction of non-sugar compounds was initially carried out using XAD-4 adsorbent resin; then, the methanol extract was purified using LLE with EtOAc. The same procedure was carried out using XAD-7, since, to our knowledge, there are not enough studies involving the use of this type of resin in honey sample treatment [12]. The extract from XAD-7 and further purification presented an improved chemical profile, as opposed to XAD-4. This is because Amberlite XAD-7, compared with other resins, is more polar with a more hydrophilic structure, while the low-energy binding process of XAD-7 allows an easier desorption of the molecules to be obtained [13]. Unfortunately, both treatments produced very low yields. To that end, sample treatment with adsorption resins was rejected, since apart from being very selective, it presents low affinity with some phenolic compounds and is a laborious and time-consuming procedure. Furthermore, high amounts of honey and solvents are required if the yield is to be satisfactory, and a combination of extraction procedures is also required. The obtained extracts were analyzed using an HPLC (Thermo Finnigan, San Jose, CA, USA) analysis (Figures S1 and S2).

Regarding LLE, EtOAc is the most common solvent used for the isolation of non-sugar compounds of honey samples [9]. Spilioti et al. performed LLE using n-BuOH and EtOAc as the second extractant solvent, which provided a higher capacity of recovering phenolic acids than other solvents [14]. Unfortunately, in this study, LLE using n-BuOH and EtOAc was not considered an appropriate choice, as n-BuOH poses certain challenges in its handling and ultimately removal from the obtained extract. Based on our results, LLE with EtOAc provided a rich chemical profile, while successfully eliminating sugars. However, the main obstacle with LLE in honey is the generation of high amounts of emulsion between the two phases, which leads to very low and mainly non-reproducible yields.

When performing extraction using CHCl_3_, vortex and centrifugation, emulsion generation is decreased, yet enough remains to interfere with the analysis. A lower amount of honey, different solvents and/or solvent systems in different ratios and different extraction periods were adopted to eliminate the emulsion (Section 2.3). Most of the obtained results presented poor chemical profiles and, as expected, very low yields, since the emulsion was not successfully eliminated. The employment of EtOAc/DCM (80/20 *v/v*) as an organic solvent system led to a significant decrease in the amount of emulsion and subsequently led to higher yields. This extraction method provided an enriched extract without the need for further purification steps; thus, it was selected as the optimal technique in this metabolite profiling approach. The extracts obtained from LLE were analyzed using ^1^H NMR spectroscopy (Figure S3).

#### 3.1.3. Data Acquisition and Processing

**Figure 1:**
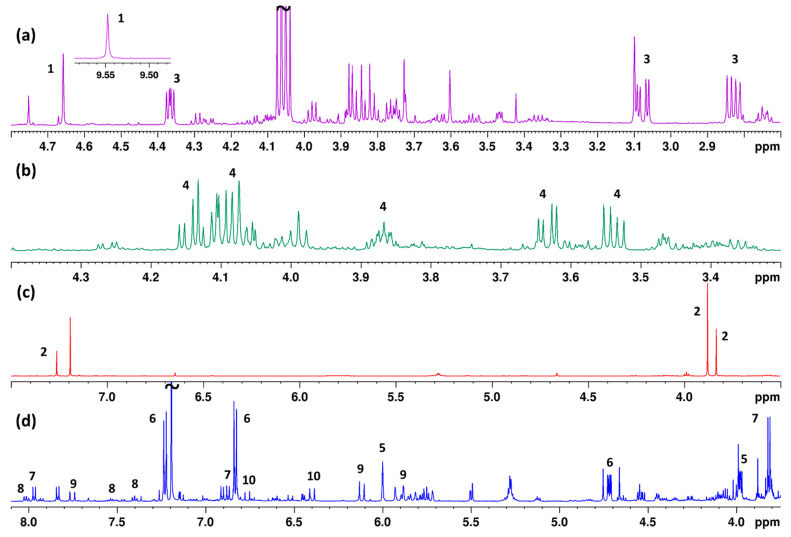
Representative 1D ^1^H NMR spectra from samples of different botanical origins: (**a**) thyme, (**b**) plant, (**c**) blossom and (**d**) heather honey. Assigned metabolites: 5-hydroxymethylfurfural (5-HMF), **1**; methyl syringate (MSYR), **2**; 3-hydroxy-4-phenyl-2-butanone, **3**; monosubstituted glycerol derivative (MG), **4**; unedone, **5**; 1-(4-methoxyphenyl)-ethane-1,2-diol, **6**; 4-methoxybenzoic acid (4-MBA), **7**; benzoic acid (BZA), **8**; abscisic acid (ABA), **9**; dehydrovomifoliol, **10**.

Over the last two decades, NMR spectroscopy has been introduced in the analyses and authentication of foods as a very reliable technique due to its speed, inherent quantitative nature and its ability to detect compounds without purification and with easy sample preparation. In the present study, each extract was dissolved in chloroform-*d* with HMDSO as the IS. A total of 152 spectra were acquired, as each sample was extracted and analyzed in duplicate, in order to increase the certainty of the method’s reliability. Representative spectra from the obtained extracts of different botanical origins are presented in Figure 1. Information about the chemical composition of the samples could be obtained, as signals corresponding to compounds of different chemical groups were discerned in each spectrum. Since the misalignment of NMR signals can take place due to several chemical and physical factors (matrix nature, pH, temperature, etc.), alignment was carried out using the icoshift tool [15].

### 3.2. Multivariate Analyses

#### 3.2.1. Classification of Honey Samples According to Their Botanical Origin

**Figure 2:**
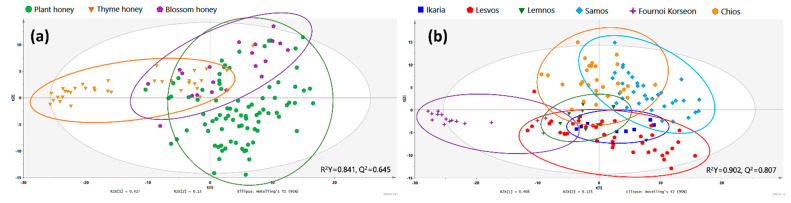
PLS-DA scores scatter plot of the dataset showing clustering based on (**a**) botanical and (**b**) geographical origin.

The dataset was first visualized by applying the PCA (Figure S4) statistical method, and observations were colored based on their botanical origin, namely, plant, thyme and blossom. Due to the limited number of samples, heather, chestnut and spring honeys were excluded from the dataset for this step. Although the PCA is an unsupervised method, a trend could be observed, as many thyme honey samples formed two separate clusters, moving away from the rest. Then, moving on to supervised statistical methods, the same dataset was used to build the respective PLS-DA model and all the possible OPLS-DA models with classes in pairs to detect any corresponding tentative biomarkers (Figure S5). In the PLS-DA model (Figure 2a), two subgroups could be detected both in the case of thyme and blossom honeys. For the former, it seemed that the separation was due to the different geographical origin, as samples in one cluster originated from the islands of Fournoi Korseon and Psara, both very small islands located in the north Aegean Sea, while in the other cluster, samples were produced in Lemnos and Ikaria. This could probably be due to the different climate and soil conditions of the first two islands compared with the latter two. Regarding the two subgroups observed in blossom honey, all samples originated from the island of Lesvos, and no other available data could explain that discrimination. In this case, other parameters were possibly involved in the observed discrimination, such as the altitude or sunshine levels of the specific subregion of Lesvos island.

As far as the OPLS-DA models are concerned, a strong discrimination between plant/thyme and blossom/thyme honey samples was revealed, except from the one between plant and blossom honey, as expected, due to their high resemblance and because neither were unifloral but mixtures of different shrubs and trees. Information about the models is summarized in Table S2.

#### 3.2.2. Classification of Honey Samples Based on Geographical Origin

As for botanical origin, a PCA was employed in the same way for the initial visualization of the classes based on the geographical origin of the samples. Due to the limited number of samples, Agios Efstratios and Psara were excluded from the dataset that was subjected to the statistical analysis. Then, the same dataset was used for the supervised analysis.

In the PCA scores scatter plot (Figure S6) formed by the first two components, an evident trend could be observed in the different classes of the dataset. Similarly to the above, a PLS-DA (Figure 2b) was used as a supervised approach to compare all groups together, while OPLS-DA models were constructed comparing the groups in pairs (Figure S7). Statistical models possessed great fitting and discrimination parameters as indicated by their descriptive and predictive values (R^2^ and Q^2^), respectively. Information about the models is summarized in Table S3. In the PLS-DA model, distinctive clusters could be clearly observed, where samples from Fournoi Korseon seemed to present the highest variation in their metabolite profile, separated by the first component. Evident clusters including samples from Lesvos, Samos and Chios were also visible. Moreover, samples from Lesvos together with the ones from Ikaria and Lemnos seemed to be separated from those from Chios and Samos.

From each OPLS-DA model created, statistically significant features were extracted from the respective S-plots and VIP lists aiming to further investigate the possible biomarkers that were responsible for the discrimination between classes.

### 3.3. Statistical Total Correlation Spectroscopy (STOCSY) and Biomarker Identification

Ιn metabolomics studies, one of the most crucial steps in the whole processing is the conversion of statistically significant features into compounds with possible ties to various quality attributes. However, this task becomes even harder in honey and NMR-based metabolite profiling due to the former’s physicochemical properties, complexity and variability and the absence of detailed databases in the latter. Therefore, a thorough workflow was followed to reveal statistically significant metabolites starting from MVA and continuing with the STOCSY statistical tool. Hence, relevant chemical shifts from the VIP lists (VIP > 1) of the OPLS-DA models were initially filtered based on their p(corr) value and then used as “driver peaks” in the STOCSY approach.

**Figure 3:**
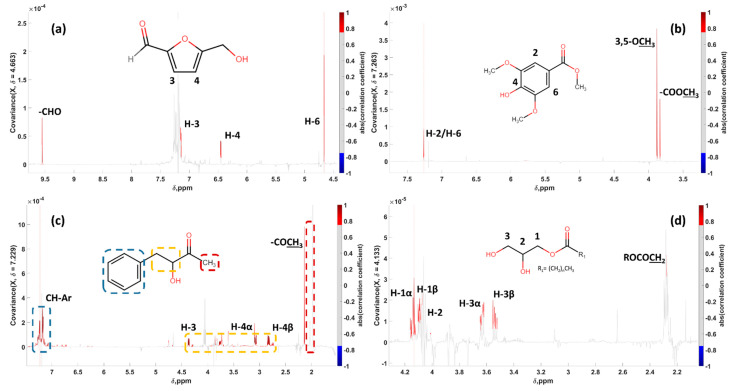
Statistical total correlation spectroscopy (STOCSY) 1D-pseudo-NMR spectra. (**a**) 5-HMF, “driver peak” at 4.663 ppm; (**b**) MSYR, “driver peak” at 7.263 ppm; (**c**) 3-hydroxy-4-phenyl-2-butanone, “driver peak” at 7.229 ppm; and (**d**) MG, “driver peak” at 4.133 ppm.

Specifically, the resonance at 4.663 ppm was used as the driver peak, revealing a pseudo-spectrum that corresponded to 5-(hydroxymethyl)furfural (5-HMF), while the resonances at 7.263, 7.229 and 4.133 ppm revealed a set of signals that corresponded to methyl syringate (MSYR), 3-hydroxy-4-phenyl-2-butanone and a monosubstituted glycerol derivative (MG), respectively (Figure 3). Detailed chemical shifts and multiplicities are enlisted in Table 2. Two-dimensional experiments (HSQC, HMBC and JRES) were additionally performed on specific total extracts to identify true or false cross-peaks correlation, thus to ensure correct structural elucidation. Additionally, the analysis of honey samples was performed using LC-HRMS to further increase the confidence in the identification of these compounds. Detailed information can be found in Tables S4 and S5.

5-HMF is a product of sugar degradation formed through Maillard reactions in foods that contain high concentrations of carbohydrates, so it can be found in many commercial products, such as whisky, wine, fruit juice, etc. [16]. Regarding honey, the Codex Alimentarius Standard has set a limit point of 40 mg/kg to ensure that the product has not been extensively heated during processing and is safe for consumption [17]. This concentration limit can vary according to the geographical origin and the temperature range of each area with a higher limit of 80 mg/kg for honey originating from tropical regions [18]. In general, 5-HMF can be characterized as a time–temperature marker for commercial products.

MSYR and 3-hydroxy-4-phenyl-2-butanone have previously been reported as constituents of honey of different botanical and geographical origins. More specifically, MSYR has been reported in asphodel honey to present good stability levels compared with fresh honey even a year after sampling. In the case of robinia, rape, chestnut, clover, linden blossom, sunflower, manuka or fir honeys, it presents very low concentrations. On the other hand, 3-hydroxy-4-phenyl-2-butanone, among others (2-hydroxy-3-pentanone, 4-methylpentanoic acid, 4-pentanoic acid), is considered as a component of honeys containing buckwheat pollen grains, while it is not present in other honey types [19,20]. Nevertheless, it is important to note that they have been also detected and identified in the volatile fraction of unifloral Greek thyme by means of ultrasound-assisted extraction via gas chromatography–mass spectrometry [21].

MGs are a general category of molecules composed by a glycerol moiety connected to a fatty acid chain through an ester bond. This class of compounds is usually found in most vegetable oils, such as coconut oil. In fact, this category of molecules appears to decrease its concentration via exposure to heat [22]. As for honey, the neutral lipids of honey samples have been isolated and identified before, with MGs being one of the major categories [23]. However, the study of the lipid fraction of honey still remains limited as a field that has to be covered and presents a very low number of scientific publications over the years.

Besides the compounds identified with the help and application of STOCSY, a dereplication method was carried out based on the literature, which allowed the identification of several other compounds in the ^1^H NMR spectra of the honeys’ total extracts to be performed. More specifically, unedone, 1-(4-methoxyphenyl)-ethane-1,2-diol, 4-methoxybenzoic acid (4-MBA), benzoic acid (BZA), abscisic acid (ABA) and dehydrovomifoliol were identified in heather honey samples. The spectral information of all the identified molecules is enlisted in Table 2.

Unedone is a sesquiterpenoid well known as a biomarker of strawberry honey and is the compound responsible for its bitter taste. Chemically, it can be characterized as an epoxidic derivative of ABA [24]. Until today, to our knowledge, there are no studies regarding the presence of unedone in heather honey, as our results indicate. In the category of alcohols, 1-(4-methoxyphenyl)-ethane-1,2-diol was isolated for the first time from the dichloromethane extract of fruiting bodies of wood-rotting fungus *Gloeophyllym odoratum* [25], and recently, its glycosylated form was isolated from the ethanol extract of *Brassica rapa* flowers [26]. There are no studies, to our knowledge, that mention its presence as a constituent of honey to this day.

BZA seems to be a common metabolite of honey; therefore, it can be found in several samples of different botanical and geographical origin, mostly by means of liquid chromatography [27]. Nevertheless, high concentrations of shikimate-pathway products, such as phenylacetic acid, BZA and 4-methoxybenzaldehyde, seem to be significant metabolites for the authentication assessment of heather honey. As mellissopalynology and sensory assessments alone were not sufficient to authenticate the honeys’ floral origin, volatile compounds from *Erica arborea* and *Calluna vulgaris* unifloral honeys were investigated. Based on the above notifications, it was proposed that the presence of BZA and decanoic acid indicated floral origin within the *Ericaceae* family, while 4-MBA was identified as a specific marker of *Erica arborea* honeys for the first time [28,29].

Nor-isoprenoids ABA and dehydrovomifoliol have been widely studied, isolated and detected in various honey samples of different botanical and geographical origins. Dehydrovomifoliol is a derivative of ABA, probably a product of degradation mainly detected in the extracts of different unifloral (chestnut, linden, orange, acacia, eucalyptus, honeydew) and polyfloral honeys [30]. Nevertheless, ABA and dehydrovomifoliol are most frequently reported in heather and strawberry honey samples [31].

### 3.4. Quality and Authentication Assessment

Quality control in honey entails two principal aspects to be examined regarding its authenticity, (i) the production procedure of the samples and (ii) the product descriptions. Regarding production procedure, honey authentication methods must be based on parameters that remain stable during processing, so that they can unveil adulteration (sugar content, moisture, amino acid profile, etc.). The determination of 5-HMF concentration is among the most important parameters, since it indicates the freshness of honeys and/or the appropriateness of the storage conditions. On the other hand, authenticity based on description is closely related to the determination of the botanical and geographical origin provided by the producers. In this regard, well-established approaches to determine the authenticity of honey include sensory, physicochemical and palynological analyses. Unfortunately, most of these approaches are time-consuming and trait specific. Thus, in the present study, a holistic, metabolite profiling approach was developed by employing the NMR analytical platform for the authenticity evaluation of honey samples in combination with chemometric tools. Attention was paid to the detection and identification of tentative biomarkers regarding the botanical or the geographical origin. The latter process, in combination with the monitoring of their relative concentrations among the different classes of the dataset, could highlight the uniqueness of the present honey samples.

#### 3.4.1. Botanical Origin

**Figure 4:**
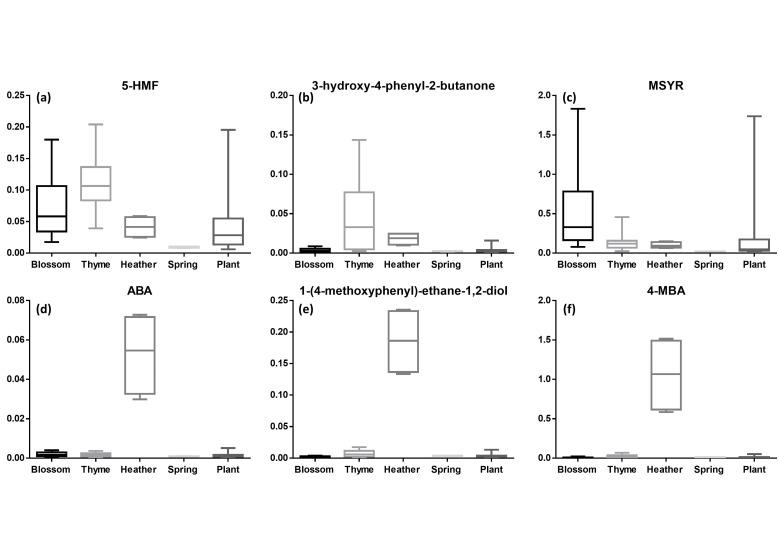
Box plots of statistically significant biomarkers based on their botanical origin are presented. (**a**) 5-HMF, (**b**) 3-hydroxy-4-phenyl-2-butanone, (**c**) MSYR, (**d**) ABA, (**e**) 1-(4-methoxyphenyl)-ethane-1,2-diol and (**f**) 4-methoxybenzoic acid. All *p*-values can be found in Table S6.

As mentioned above, 5-HMF is used as an indicator for honey’s freshness and quality. It has been extensively studied, and while there are studies proving that this compound is not detected in freshly collected honeys, others have demonstrated its existence in unprocessed honey in small quantities [39,40]. Based on the constructed box plots (Figure 4), in the present study, 5-HMF could be found in most samples. However, the highest concentrations were detected in blossom and thyme honey samples. In regards to blossom honey samples, there was no information regarding their exact botanical origin, so there was no possibility of drawing reliable conclusions. In regards to thyme honey, the latter is considered as a unifloral blossom honey; our results are in accordance with the literature, as it has previously been reported that thyme honey possesses elevated concentrations of 5-HMF, due to its higher concentration of fructose compared with glucose [41]. Monofloral honeys in general have a higher concentration of fructose instead of glucose [42]. Since 5-HMF seems to be selectively produced from keto-hexoses such as fructose [18], it is expected to be present at higher concentrations in these types of honey.

On the contrary, plant honey seems to have a lower concentration of 5-HMF, while being absent in spring honey. Past studies mention that honeydew honeys contain lower concentrations of monosaccharides and are characterized by high concentrations of oligosaccharides [42]. As mentioned above, fructose is the main precursor of 5-HMF. Consequently, the lower concentration of monosaccharides in plant honeys led to lower concentrations of 5-HMF. Additionally, even though there was no information about the exact botanical origin of spring honey, we could suggest that most of these samples corresponded to honeydew honey and not to honey from flower nectar. Nevertheless, the low number of spring honey samples led to an ambiguous outcome in this regard.

A different pattern could be observed for MSYR (Figure 4). This compound has been determined as a biomarker of Italian asphodel honey [19]. Based on the respective box plots, blossom honey presented a high relative concentration of MSYR compared with thyme and other honey samples. This was opposed to a past study, where it was detected at high concentrations in Greek thyme honey [21]. Even though thyme honey is also considered as blossom honey, MSYR was found at very low concentrations in these honey samples. This could possibly suggest that it could be considered as a floral honey biomarker other than a thyme honey one. However, it is worth mentioning that the group of blossom honey presented additionally high variation within the group itself, as it is obvious in Figure 4. So, close attention should be taken when considering any suggestions made here.

Regarding 3-hydroxy-4-phenyl-2-butanone, it was mainly found in thyme honey samples at relatively high concentrations. This is in accordance with the literature, since it has previously been reported at high concentrations in the volatile composition of Greek thyme honeys [43]. Therefore, it could be considered as an important biomarker of unifloral Greek thyme honey.

In the case of 1-(4-methoxyphenyl)-ethane-1,2-diol, ABA and 4-MBA, the highest concentrations of all three compounds were found in heather honey, as they were almost absent in all other honey samples of different botanical origins (Figure 4). However, while this is a significant finding, the low number of heather samples suggests the need of further efforts towards verifying this observation. For the latter two, this is in compliance with the literature, since, as already mentioned, ABA and 4-MBA have been extensively determined as biomarkers of heather honey [31]. Concerning 1-(4-methoxyphenyl)-ethane-1,2-diol, there are studies where its presence was determined in fungi and *Brassica* flowers [25,26], but there are no previous studies identifying it as a honey constituent. This is the first time that 1-(4-methoxyphenyl)-ethane-1,2-diol is mentioned in honey samples to our knowledge.

BZA and dehydrovomifoliol were both found to be at higher concentrations in heather honey, as expected [29]. It has previously been mentioned that shikimate-pathway products are known biomarkers of heather honey [28]; additionally, BZA is an important marker of the *Ericaceae* family [29]. As for dehydrovomifoliol, it has already been identified as a marker of heather honey [31]. However, in the case of BZA, thyme and spring honeys are in second place, after plant and blossom honeys, while for dehydrovomifoliol, this gap appears to have closed significantly. As for MG, it was detected mostly in thyme honey and at lower concentrations in spring and heather honey (Figure S8). Finally, unedone, a well-known marker of strawberry honey [24], was identified here for the first time as a constituent of heather honey and in significant amounts (Figure S8). Honeys with different botanical origin in this study were characterized by very low relative concentrations, indicating that unedone could also be considered as a biomarker of heather honey. However, this observation requires the analysis of a larger number of samples of this specific botanical origin in order to ensure the validity of the results.

#### 3.4.2. Geographical Origin

As far as geographical origin is concerned, any observations made in this study cannot be either confirmed or disproved due to the absence of any relative literature to our knowledge. Samples from the islands of Ikaria, Lemnos, Fournoi and Psara were initially compared, as the botanical origin of honeys from these islands is solely thyme. Another comparison was conducted amongst the islands of Lesvos, Samos and Chios, including only plant honey samples as botanical origin.

**Figure 5:**
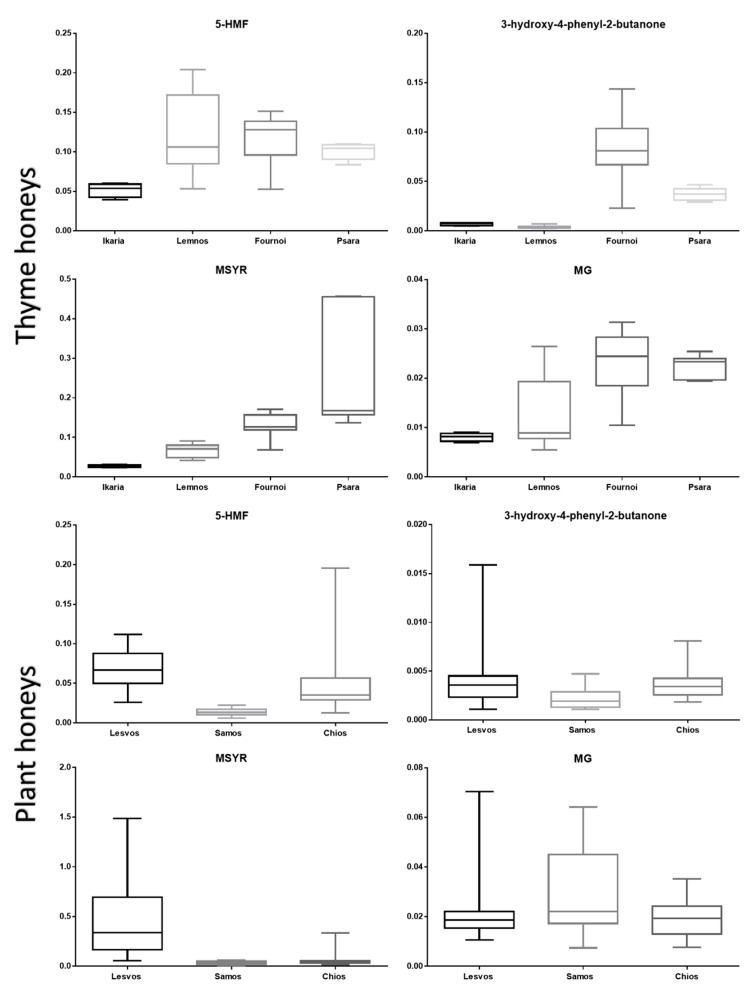
Box plots of statistically significant biomarkers based on their geographical origin (*p*-value ≤ 0.05 was considered as statistically significant). All *p*-values can be found in Tables S7 and S8.

The data displayed in the box plot below concerning thyme honeys (Figure 5) show that 5-HMF could be found at higher levels in samples from Lemnos, Fournoi and Psara than in samples from Ikaria. However, among the former three islands no variations could be observed. In the case of 3-hydroxy-4-phenyl-2-butanone (Figure 5), aside from being a possible indicator of botanical origin as already mentioned, it seems that geographical origin could also play an important role in its concentration levels in honey. Specifically, thyme honeys from the island of Fournoi showed significantly higher levels of this compound, followed by the island of Psara, with Ikaria and Lemnos laying on similarly low grounds. This compound could, therefore, be considered as a geographical marker of thyme honeys in favor of the island of Fournoi. On the other hand, MSYR (Figure 5) appeared to be present at high concentrations in samples coming from Psara, when compared with the islands of Fournoi and Lemnos, while it was almost absent in samples coming from Ikaria. In the case of the MG derivative (Figure 5), Psara and Fournoi outperformed Lemnos and Ikaria. This could possibly imply that this MG derivative can be considered as a geographical biomarker in favor of the former two islands.

Finally, when examining the honey samples from Lesvos, Samos and Chios of plant origin, 5-HMF could be found at higher levels in samples from Lesvos, followed by Chios and then Samos. A similar pattern was observed for 3-hydroxy-4-phenyl-2-butanone, with Lesvos and Chios honeys both having slightly higher concentrations than Samos honeys. On the contrary, MSYR seemed to be present at notably high levels in samples from Lesvos, while plant honey samples from Samos and Chios seems to be characterized by the absence of this compound. Lastly, regarding the MG derivative, it seemed that plant honey samples coming from Samos could be distinguished by the higher levels of the specific metabolite, while samples from Lesvos and Chios did not present a significant difference among them.

## 4. Conclusions

In this study, NMR-based metabolite profiling in combination with multivariate and univariate data analyses was applied for the first time in non-sugar fractions of Greek honey samples. It seemed to be a fast and reliable methodology for honey quality control and authenticity assessment. Moreover, STOCSY proved to be a valuable additional statistical tool for biomarker identification, with the present being its first application in honey samples. Different compounds were identified as biomarkers of the botanical and geographical origin of the samples, respectively. 5-HMF and 3-hydroxy-4-phenyl-2-butanone were found to be valuable biomarkers of thyme honey, with the latter being also an Important marker of thyme honeys specifically from the island of Fournoi. In this study, blossom honeys were characterized by high levels of MSYR, as were plant honeys from the island of Lesvos. The MG derivative could be primarily used as a possible biomarker of plant honeys with Samos as geographical origin and the islands of Fournoi and Psara for honey samples of thyme botanical origin. Compounds such as ABA, dehydrovomifoliol, BZA and 4-MBZA were found and identified in heather honey samples, in accordance with the previous literature. Unedone, an important biomarker of strawberry honey, was identified here as a biomarker of heather honey for the first time. Finally, 1-(4-methoxyphenyl)-ethane-1,2-diol, a natural compound detected to this day in fungi and *Brassica* flowers, was identified as a honey constituent for the first time. Nevertheless, further studies of bigger datasets are necessary to proceed to solid conclusions regarding specific biomarkers related to their botanical origin and especially regarding their geographical origin.

## Figures and Tables

**Table 1:** List of analyzed honey samples along with their metadata (classification).

Geographical Origin	Geographical Coordinates	Botanical Origin/Variety	Sample Size
Agios Efstratios	39°31′8″ N, 25°0′27″ E	Plant	1
Ikaria	37°35′58″ N, 26°10′0″ E	Spring honey/Heather	6
Lesvos	39°10′58″ N, 26°12′10″ E	Chestnut/Blossom/Plant	21
Lemnos	39°54′10″ N, 25°13′13″ E	Thyme	5
Samos	37°43′41″ N, 26°49′10″ E	Plant	17
Fournoi Korseon	37°35′27″ N, 26°30′8″ E	Thyme	8
Chios	38°22′59″ N, 26°2′40″ E	Plant	15
Psara	38°34′10″ N, 25°35′5″ E	Thyme	3

**Table 2:** ^1^H NMR spectroscopic data (600 MHz; chloroform-*d*) of identified compounds in honey samples.

No.	Compound	*δ*1H (Multiplicity, *J* in Hz, Assignment)	*δ*13C Assignment	Reference
1	5-HMF	9.58 (s, CHO), 7.21 (d, *J* = 3.6 Hz, H-3), 6.51 (d, *J* = 3.5 Hz, H-4), 4.71 (s, -CH_2_OH)	177.8 (CHO), 160.4(C-4), 122.0 (C-3), 110.1 (C-2), 57.9 (CH_2_OH)	[32]
2	MSYR	7.31 (s, H-2/H-6), 3.96 (s, 3/5-OCH_3_), 3.91 (s, COOCH_3_)	166.7 (C=O), 146.6 (C-3/5), 139.3 (C-4), 121.1 (C-1), 107.0 (C-2/6), 56.6 (3/5-OCH_3_), 52.0 (COOCH_3_),	[33]
3	3-hydroxy-4-phenyl-2-butanone	4.43 (dd, *J* = 4.80/7.30 Hz, H-3), 3.14 (dd, *J* = 4.7/ 14.22 Hz, H-4β), 2.89 (dd, *J* = 7.30/14.26 Hz, H-4α), 2.20, (s, COOCH_3_)	208.9 (C=O), 136.4 (C-1′), 129.2 (C-2′), 77.9 (C-3), 40.2 (C-4), 26.2 (C-1)	[34]
4	MG	4.21 (dd, *J* = 4.64/11.59 Hz, H-1α), 4.15 (dd, *J* = 5.87/11.09 Hz, H-1β), 3.93 (m, H-2), 3.69 (dd, *J* = 4.08/11.37 Hz, H-3α), 3.60 (dd, *J* = 5.72/11.39 Hz, H-3β)	173.9 (COO), 79.5 (C-2), 65.3 (C-1), 63.5 (C-3), 34.2 (C-1′), 29.3 (C-2′)	[35]
5	Unedone	6.03 (br s, H-4), 4.04 (dd, *J* = 3.92/8.73 Hz, H-8), 3.87 (m, H-9), 3.13 (d, *J* = 8.69 Hz, H-7), 2.50/2.38 (dd, *J* = 17/1.2 Hz, H-2α/β), 1.81 (d, *J* = 1.2 Hz, H-13), 1.28 (d, *J* = 6.47 Hz, H-10), 1.19 (s, H-11), 1.01 (s, H-12)	197.0 (C=O), 161.2 (C-5), 129.6 (C-4), 72.4 (C-8), 69.2 (C-9), 68.7 (C-6), 66.0 (C-7), 51,2 (C-2), 37.1 (C-1), 26.9 (C-12), 25.7 (C-11), 19.7 (C-10), 18.0 (C-13)	[24]
6	1-(4-methoxyphenyl)-ethane-1,2-diol	7.28 (d, *J* = 8.65 Hz, H-2’/6′), 6.89 (d, *J* = 8.72 Hz, H-3′/5′), 4.78 (dd, *J*= 3.63/ 8.18 Hz, H-1), 3.80 (s, OCH_3_), 3.73 (dd, *J* = 3.70/ 11.34 Hz, H-2β), 3.66 (dd, *J* = 8.14/ 11.28 Hz, H-2α)	159. 4 (C-4′), 132.6 (C-1′), 127.7 (C-2′/6′), 114.3 (C-3′/5′), 74.7 (C-1), 68.1 (C-2), 55.4 (OCH_3_)	[36]
7	4-MBA	8.03 (d, *J* = 8.99 Hz, H-2/6), 6.93 (d, *J* = 8.98 Hz, H-3/5), 3.87 (s, OCH_3_)	170.3 (COOH), 164.0 (C-4), 132.5 (C-2/6), 114.3 (C-3/5), 55.4 (OCH_3_)	[37]
8	BZA	8.08 (dd, *J* = 1.35/8.31 Hz, H-2/6), 7.60 (t, *J* = 7.32 Hz, H-4), 7.46 (t, *J* = 7.60 Hz, H-3/5)	170.4 (COOH), 133.9 (C-4), 130.4 (C-2/6), 130.2 (C-1), 128.8 (C-3/5)	[37]
9	ABA	7.81 (d, *J* = 16.09 Hz, H-4), 6.18 (d, *J* = 16 Hz, H-5), 5.94 (s, H-8), 5.78 (s, H-2), 2.48–2.32 (m, H-10α/β), 2.08 (s, H-15), 1.89 (s, H-14), 1.11 (s, H-12), 1.01 (s, H-13)	199.1 (C=O), 151.8 (C-7)136.9 (C-5), 128.5 (C-4), 127.6 (C-8), 117.8 (C-2), 80.1 (C-6), 50.0 (C-10), 41.8 (C-11), 23.0 (C-12/13), 21.8 (C-15), 19.26 (C-14)	[30]
10	Dehydrovomifoliol	6.83 (d, *J* = 15.75 Hz, H-4), 6.46 (d, *J* = 15.82 Hz, H-3), 5.94 (s, H-7), 2.48–2.32 (m, H-9α/β), 2.30 (s, H-1), 1.89 (s, H-13), 1.11 (s, H-11), 1.01 (s, H-12)	199.0 (8-C=O) 197.4 (2-C=O), 151.8 (C-6), 145.4 (C-4), 130.8 (C-3), 127.6 (C-7), 79.64 (C-5), 50.0 (C-9), 41.8 (C-10), 28.6 (C-1), 23.0 (C-11/12), 19.2 (C-13)	[38]

5-HMF, 5-(hydroxymethyl)furfural; MSYR, methyl syringate; MG, mono-substituted glycerol derivative; 4-MBA, 4-methoxybenzoic acid; BZA, benzoic acid; ABA, abscisic acid. br s, broad singlet; d, doublet; dd, doublet of doublets; m, multiplet; s, singlet; t, triplet.

## Data Availability

The data are available from the corresponding author.

## Supplementary Materials

The following supporting information can be downloaded at: https://www.mdpi.com/article/10.3390/foods11182853/s1, Figure S1: HPLC chromatograms of thyme honey using Amberlite XAD-4 adsorbent resin: (a) before LLE and (b) after LLE at 290 nm (green), 340 nm (blue) and 360 nm (black), Figure S2: HPLC chromatograms of thyme honey using Amberlite XAD-7 adsorbent resin: (a) before LLE and (b) after LLE at 290 nm (green), 340 nm (blue) and 360 nm (black), Figure S3: Representative 1H NMR 600 MHz spectra obtained when using a biphasic system: (a) DCM/MeOH 90:10 *v/v*, (b) EtOAc/DCM 80:20 *v/v*, (c) EtOAc/DCM 90:10 *v/v* and (d) EtOAc 100%. Spectral intensity of region 10–5.7 ppm was 64-fold higher than that of region 5.5–0.5 ppm, Figure S4: PCA scores scatter plot of the dataset showing clustering based on botanical origin. Heather, chestnut and spring honeys were excluded due to their limited sample size, Figure S5: OPLS-DA scores scatter plots based on botanical origin with their respective S-plots: (a) plant vs. thyme, (b) plant vs. blossom, (c) thyme vs. blossom, Figure S6: PCA scores scatter plot of the dataset showing clustering based on geographical origin. Psara and Agios Efstratios honeys were excluded due to their limited sample size, Figure S7: OPLS-DA scores scatter plots based on geographical origin with their respective S-plots, Figure S8: Box plots of statistically significant biomarkers based on their botanical origin are presented, Table S1: Metadata of all the honey samples from the northeastern Aegean region of Greece under study, Table S2: List of models based on botanical origin. A, number of components; N, number of observations; cumulative R^2^X, R^2^Y, Q^2^ values, F statistic and *p*-values determined using CV-ANOVA, Table S3: List of models based on geographical origin. A, number of components; N, number of observations; cumulative R^2^X, R^2^Y, Q^2^ values, F statistic and *p*-values determined using CV-ANOVA, Table S4: Identified compounds using HRMS and HRMS/MS in negative ionization mode, Table S5: Identified compounds using HRMS and HRMS/MS in positive ionization mode, Table S6: Box plot *p*-values comparing all the possible groups based on botanical origin. Statistically significant, *p*-value ≤ 0.05, Table S7: Box plot *p*-values comparing thyme honey samples of different geographical origin. Statistically significant, *p*-value ≤ 0.05, Table S8: Box plot *p*-values comparing plant honey samples of different geographical origin. Statistically significant, *p*-value ≤ 0.05.

## References

[1] Consonni R., Cagliani L.R., Cogliati C. (2012). NMR Characterization of Saccharides in Italian Honeys of Different Floral Sources. J. Agric. Food Chem..

[2] Se K.W., Wahab R.A., Syed Yaacob S.N., Ghoshal S.K. (2019). Detection Techniques for Adulterants in Honey: Challenges and Recent Trends. J. Food Compos. Anal..

[3] Camina J.M., Pellerano R.G., Marchevsky E.J. (2012). Geographical and Botanical Classification of Honeys and Apicultural Products by Chemometric Methods. A Review. Curr. Anal. Chem..

[4] Ohmenhaeuser M., Monakhova Y.B., Kuballa T., Lachenmeier D.W. (2013). Qualitative and Quantitative Control of Honeys Using NMR Spectroscopy and Chemometrics. ISRN Anal. Chem..

[5] Tsagkaris A.S., Koulis G.A., Danezis G.P., Martakos I., Dasenaki M., Georgiou C.A., Thomaidis N.S. (2021). Honey Authenticity: Analytical Techniques, State of the Art and Challenges. RSC Adv..

[6] Cloarec O., Dumas M.-E., Craig A., Barton R.H., Trygg J., Hudson J., Blancher C., Gauguier D., Lindon J.C., Holmes E. (2005). Statistical Total Correlation Spectroscopy: An Exploratory Approach for Latent Biomarker Identification from Metabolic 1 H NMR Data Sets. Anal. Chem..

[7] Beteinakis S., Papachristodoulou A., Gogou G., Katsikis S., Mikros E., Halabalaki M. (2020). NMR-Based Metabolic Profiling of Edible Olives-Determination of Quality Parameters. Molecules.

[8] Kazalaki A., Misiak M., Spyros A., Dais P. (2015). Identification and Quantitative Determination of Carbohydrate Molecules in Greek Honey by Employing 13C NMR Spectroscopy. R. Soc. Chem..

[9] Pyrzynska K., Biesaga M. (2009). Analysis of Phenolic Acids and Flavonoids in Honey. TrAC Trends Anal. Chem..

[10] Schievano E., Peggion E., Mammi S. (2010). 1H Nuclear Magnetic Resonance Spectra of Chloroform Extracts of Honey for Chemometric Determination of Its Botanical Origin. J. Agric. Food Chem..

[11] Pascual-Maté A., Osés S.M., Fernández-Muiño M.A., Sancho M.T. (2018). Analysis of Polyphenols in Honey: Extraction, Separation and Quantification Procedures. Sep. Purif. Rev..

[12] Tomás-Barberán F.A., Blázquez M.A., Garcia-Viguera C., Ferreres F., Tomás-Lorente F. (1992). A Comparative Study of Different Amberlite XAD Resins in Flavonoid Analysis. Phytochem. Anal..

[13] Moore R.A., Karasek F.W. (1984). Extraction of Organic Compounds from Aqueous Media by Amberlite XAD Resins. Int. J. Environ. Anal. Chem..

[14] Spilioti E., Jaakkola M., Tolonen T., Lipponen M., Virtanen V., Chinou I., Kassi E., Karabournioti S., Moutsatsou P. (2014). Phenolic Acid Composition, Antiatherogenic and Anticancer Potential of Honeys Derived from Various Regions in Greece. PLoS ONE.

[15] Savorani F., Tomasi G., Engelsen S.B. (2010). Icoshift: A Versatile Tool for the Rapid Alignment of 1D NMR Spectra. J. Magn. resonn..

[16] Kew W., Goodall I., Uhrín D. (2019). Analysis of Scotch Whisky by 1H NMR and Chemometrics Yields Insight into Its Complex Chemistry. Food Chem..

[17] (2019). WHO and FAO STANDARD FOR HONEY CXS 12-1981. Codex Aliment..

[18] Shapla U.M., Solayman M., Alam N., Khalil M.I., Gan S.H. (2018). 5-Hydroxymethylfurfural (HMF) Levels in Honey and Other Food Products: Effects on Bees and Human Health. Chem. Cent. J..

[19] Tuberoso C.I.G., Bifulco E., Jerkovic I., Caboni P., Cabras P., Floris I. (2009). Methyl Syringate: A Chemical Marker of Asphodel (Asphodelus Microcarpus Salzm. et Viv.) Monofloral Honey. J. Agric. Food Chem..

[20] Wolski T., Tambor K. (2006). Identification of Honey Volatile Components by Solid Phase Microextraction (SPME) and Gas Chromatography/Mass Spectrometry (GC/MS). J. Apic. Sci..

[21] Alissandrakis E., Tarantilis P.A., Pappas C., Harizanis P.C., Polissiou M. (2009). Ultrasound-Assisted Extraction Gas Chromatography-Mass Spectrometry Analysis of Volatile Compounds in Unifloral Thyme Honey from Greece. Eur. Food Res. Technol..

[22] Dayrit F.M., Buenafe O.E.M., Chainani E.T., de Vera I.M.S. (2008). Analysis of Monoglycerides, Diglycerides, Sterols, and Free Fatty Acids in Coconut (*Cocos Nucifera* L.) Oil by 13 P NMR Spectroscopy. J. Agric. Food Chem..

[23] Kapoulas V.M., Mastronicolis S.K., Galanos D.S. (1977). Identification of the Lipid Components of Honey. Z. Lebensm. Unters.—Forsch..

[24] Tuberoso C.I.G., Bifulco E., Caboni P., Cottiglia F., Cabras P., Floris I. (2010). Floral Markers of Strawberry Tree (*Arbutus Unedo* L.) Honey. J. Agric. Food Chem..

[25] Rösecke J., König W.A. (2000). Constituents of Various Wood-Rotting Basidiomycetes. Phytochemistry.

[26] Zhao C.C., Shen J., Chen J., Shao J.H., Li K.H., Gu W.Y., Miao B.J. (2019). Phenolic Glycoside Constituents from Brassica Rapa Flowers and Their α-Glucosidase Inhibitory Activity. Nat. Prod. Res..

[27] Ciulu M., Spano N., Pilo M.I., Sanna G. (2016). Recent Advances in the Analysis of Phenolic Compounds in Unifloral Honeys. Molecules.

[28] Castro-Vázquez L., Díaz-Maroto M.C., Pérez-Coello M.S. (2007). Aroma Composition and New Chemical Markers of Spanish Citrus Honeys. Food Chem..

[29] Guyot C., Scheirman V., Collin S. (1999). Floral Origin Markers of Heather Honeys: Calluna Vulgaris and Erica Arborea. Food Chem..

[30] Schievano E., Morelato E., Facchin C., Mammi S. (2013). Characterization of Markers of Botanical Origin and Other Compounds Extracted from Unifloral Honeys. J. Agric. Food Chem..

[31] Machado A.M., Miguel M.G., Vilas-Boas M., Figueiredo A.C. (2020). Honey Volatiles as a Fingerprint for Botanical Origin—a Review on Their Occurrence on Monofloral Honeys. Molecules.

[32] Phan H.B., Thi Nguyen Q.B., Luong C.M., Tran K.N., Tran P.H. (2021). A Green and Highly Efficient Synthesis of 5-Hydroxymethylfurfural from Monosaccharides Using a Novel Binary Ionic Liquid Mixture. Mol. Catal..

[33] Xian Y., Zhou H., Wang X., Yu J., Zheng Z., Yang B. (2014). Chemical Constituents of Gleditsia Sinensis Thorns. Asian J. Chem..

[34] Gocke D., Nguyen C.L., Pohl M., Stillger T., Walter L., Müller M. (2007). Branched-Chain Keto Acid Decarboxylase from Lactococcus Lactis (KdcA), a Valuable Thiamine Diphosphate-Dependent Enzyme for Asymmetric C-C Bond Formation. Adv. Synth. Catal..

[35] Batovska D.I., Tsubota S., Kato Y., Asano Y., Ubukata M. (2004). Lipase-Mediated Desymmetrization of Glycerol with Aromatic and Aliphatic Anhydrides. Tetrahedron Asymmetry.

[36] Cazetta T., Moran P.J.S., Rodrigues J.A.R. (2014). Highly Enantioselective Deracemization of 1-Phenyl-1,2-Ethanediol and Its Derivatives by Stereoinversion Using Candida Albicans in a One-Pot Process. J. Mol. Catal. B Enzym..

[37] Sathyanarayana P., Ravi O., Muktapuram P.R., Bathula S.R. (2015). Copper Catalyzed Oxygen Assisted C(CNOH)-C(Alkyl) Bond Cleavage: A Facile Conversion of Aryl/Aralkyl/Vinyl Ketones to Aromatic Acids. Org. Biomol. Chem..

[38] Kisiel W., Michalska K., Szneler E. (2004). Norisoprenoids from Aerial Parts of Cichorium Pumilum. Biochem. Syst. Ecol..

[39] Kukurová K., Karovičová J., Greif G., Kohajdová Z., Lehkoživová J. (2006). Determination of 5-Hydroxymethylfurfural after Winkler and by the HPLC Method for Authentication of Honey. Chem. Pap..

[40] Chakir A., Romane A., Marcazzan G.L., Ferrazzi P. (2016). Physicochemical Properties of Some Honeys Produced from Different Plants in Morocco. Arab. J. Chem..

[41] Tsiapara A.V., Jaakkola M., Chinou I., Graikou K., Tolonen T., Virtanen V., Moutsatsou P. (2009). Bioactivity of Greek Honey Extracts on Breast Cancer (MCF-7), Prostate Cancer (PC-3) and Endometrial Cancer (Ishikawa) Cells: Profile Analysis of Extracts. Food Chem..

[42] Siddiqui I.R. (1970). The Sugars of Honey. Adv. Carbohydr. Chem. Biochem..

[43] Alissandrakis E., Tarantilis P.A., Harizanis P.C., Polissiou M. (2007). Comparison of the Volatile Composition in Thyme Honeys from Several Origins in Greece. J. Agric. Food Chem..

