# Supplementary material for: NMR Metabolite Profiling in the Quality and Authentication Assessment of Greek Honey—Exploitation of STOCSY for Markers Identification
Source: Foods. 2022 Sep 15;11(18):2853. doi: 10.3390/foods11182853 (PMC9498239; doi:10.3390/foods11182853)
Supplement: Supplementary file 1 [file foods-11-02853-s001.zip › foods-1889146-supplementary.pdf]

Supplementary Materials

Article

NMR Metabolite Profiling in the Quality and Authentication Assessment of Greek Honey – Exploitation of STOCSY for Markers Identification

Gabriela Belén Lemus Ringele [1], Stavros Beteinakis [1], Anastasia Papachristodoulou [1], Evangelos Axiotis [1,2], Emmanuel Mikros [3] and Maria Halabalaki [1,*]

[1] Division of Pharmacognosy and Natural Products Chemistry, Department of Pharmacy, National and Kapodistrian University of Athens, Panepistimiopolis, Zografou, 15771 Athens, Greece

[2] Natural Products Research Center "NatProAegean", Gera, 81106 Lesvos, Greece

[3] Division of Pharmaceutical Chemistry, Department of Pharmacy, National and Kapodistrian University of Athens, Panepistimiopolis, Zografou, 15771 Athens, Greece

* Correspondence: mariahal@pharm.uoa.gr

Table S*1*. Metadata of all the honey samples from the Northeast Aegean region of Greece under study.

Sample Code	Geographical Origin (Region)	Subregion	Botanical Origin
HON01	Agios Efstratios	Agios Efstratios	Plant
HON02	Ikaria	Gialiskari	Spring
HON03	Ikaria	Gialiskari	Spring
HON04	Ikaria	Gialiskari	Heather
HON05	Ikaria	Gialiskari	Heather
HON06	Ikaria	Gialiskari	Thyme
HON07	Ikaria	Gialiskari	Thyme
HON08	Lesvos	Agiasos	Chestnut
HON09	Lesvos	Agra	Blossom
HON10	Lesvos	Airport	Blossom
HON11	Lesvos	Brisa	Blossom
HON12	Lesvos	Gera	Blossom
HON13	Lesvos	Eresos/Sigkri	Blossom
HON14	Lesvos	Thermis	Blossom
HON15	Lesvos	Kalloni	Blossom
HON16	Lesvos	Kapi	Plant
HON17	Lesvos	Kapi	Plant
HON18	Lesvos	Loutra/Mytilene	Blossom
HON19	Lesvos	Mesotopos	Plant
HON20	Lesvos	Mesotopos	Plant
HON21	Lesvos	Plagia Plomariou	Plant

**Sample Code**	**Geographical Origin (Region)**	**Subregion**	**Botanical Origin**
HON22	Lesvos	Plagia Plomariou	Plant
HON23	Lesvos	Plomari	Blossom
HON24	Lesvos	Plomari	Plant
HON25	Lesvos	Plomari	Plant
HON26	Lesvos	Stypsi	Blossom
HON27	Lesvos	Plagia Plomariou	Plant
HON28	Lesvos	Plagia Plomariou	Plant
HON29	Lemnos	Agia Marina	Thyme
HON30	Lemnos	Kaspakas	Thyme
HON31	Lemnos	Manikati	Thyme
HON32	Lemnos	Chavouli	Thyme
HON33	Lemnos	Agia Marina	Thyme
HON34	Samos	Agios Ioannis	Plant
HON35	Samos	Drakei	Plant
HON36	Samos	Kallithea	Plant
HON37	Samos	Kampos	Plant
HON38	Samos	Koutakaika	Plant
HON39	Samos	Manolates	Plant
HON40	Samos	Mytileneoi	Plant
HON41	Samos	Paradeika	Plant
HON42	Samos	Paradeika	Plant
HON43	Samos	Petalouda	Plant
HON44	Samos	Platanos	Plant
HON45	Samos	Platanos	Plant
HON46	Samos	Sakouleika	Plant
HON47	Samos	Stavrinides	Plant
HON48	Samos	Stavrinides	Plant
HON49	Samos	Idroussa	Plant
HON50	Samos	Charoupies	Plant
HON51	F. Korseon	Fournoi Korseon	Thyme
HON52	F. Korseon	Fournoi Korseon	Thyme
HON53	F. Korseon	Kamari	Thyme
HON54	F. Korseon	Kamari	Thyme
HON55	F. Korseon	Bali	Thyme
HON56	F. Korseon	Kamari	Thyme
HON57	F. Korseon	Bali	Thyme
HON58	F. Korseon	Kamari	Thyme
HON59	Chios	Chios	Plant
HON60	Chios	Chios	Plant
HON61	Chios	Aygonyma	Plant
HON62	Chios	Vessa	Plant
HON63	Chios	Viki	Plant
HON64	Chios	Viki	Plant
HON65	Chios	Egrigyros	Plant

Sample Code	Geographical Origin (Region)	Subregion	Botanical Origin
HON66	Chios	Tholopotami	Plant
HON67	Chios	Kabi	Plant
HON68	Chios	Kardamyla	Plant
HON69	Chios	Kardamyla	Plant
HON70	Chios	Kourounia	Plant
HON71	Chios	Leptypoda	Plant
HON72	Chios	Spartounda	Plant
HON73	Chios	Chalandra	Plant
HON74	Psara	Psara	Thyme
HON75	Psara	Psara	Thyme
HON76	Psara	Psara	Thyme

High-performance liquid chromatography (HPLC-DAD)

An analytical HPLC (Thermo Finnigan system) consisting of a UV detector Spectra System UV2000LP detector; Diode-Array Detection (DAD) SpectraSystem UV6000LP a P4000 pump, an AS3000 automatic sample injector and a 1000 degasser (all SpectraSystem), was used for qualitative analysis of honey samples. All samples were prepared in a concentration of 10 mg/mL. Chromatograms were obtained using a Supelco RP18 UniverSilHS column (250x4.6mm, 5 um), with a mobile phase consisted of Solvent A (H_2O+0.1%FA *v/v*) and Solvent B (Acetonitrile, ACN). Injection volume was set at 20 uL, and the analysis was conducted at room temperature. The gradient elution method used is described in the table below. Data acquisition was performed using ChromQuestTM Software 4.1.

Time (min)	Solvent A (H2O+0.1%FA)	Solvent B (ACN)	Flow (mL/min)
0	85	15	1
15	70	30	1
30	60	40	1
35	60	40	1
40	50	50	1
45	50	50	1
55	10	90	1
70	10	90	1
73	85	15	1
75	85	15	1

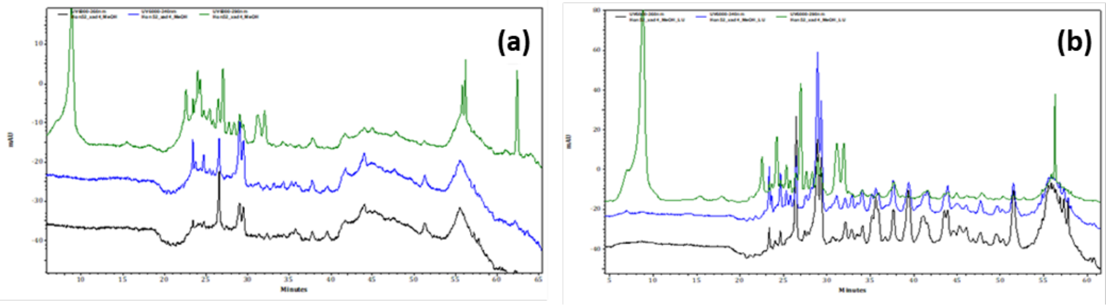

Figure S1. HPLC chromatograms of a Thyme honey using Amberlite XAD-4 adsorbent resin: (**a**) Before LLE and (**b**) after LLE at 290 nm (green), 340 nm (blue) and 360 nm (black).

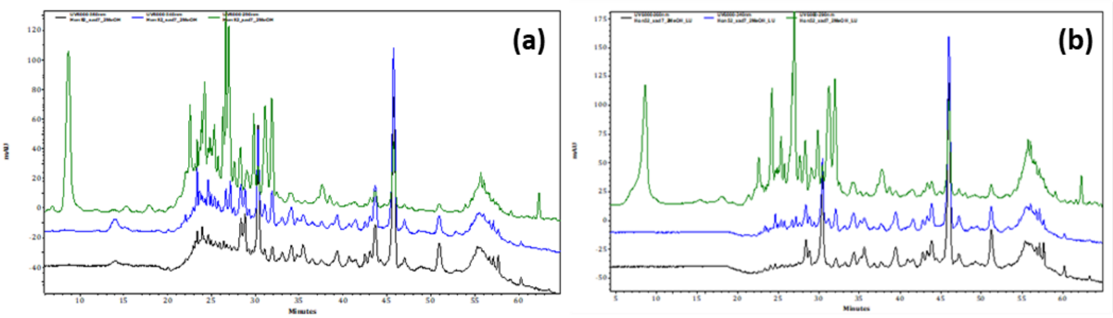

Figure S2. HPLC chromatograms of a Thyme honey using Amberlite XAD-7 adsorbent resin: (**a**) Before LLE and (**b**) after LLE at 290 nm (green), 340 nm (blue) and 360 nm (black).

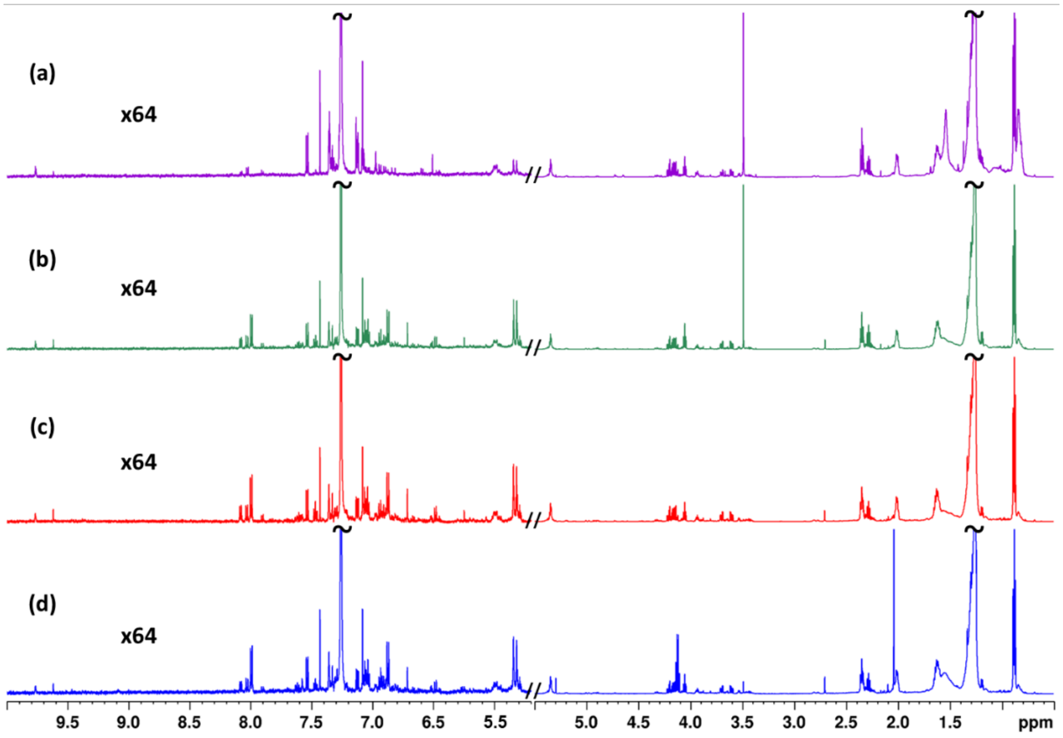

Figure S3. Representative 1H NMR 600 MHz spectra obtained when using as biphasic system: (**a**) DCM/MeOH 90:10 *v*/*v*, (b) EtOAc/DCM 80:20 *v*/*v*, (**c**) EtOAc/DCM 90:10 *v*/*v* and (**d**) EtOAc 100%. Spectral intensity for the region between 10-5.7 ppm is 64-fold higher than the region between 5.5-0.5 ppm.

Table S2. List of models based on botanical origin. A, number of components; N, number of observations; cumulative R^2X, R^2Y, Q^2 values, F statistic and p-values determined from CV-ANOVA.

Model	A	N	R^2X	R^2Y	Q^2	F	p-value
PCA	10	142	0.795		0.735		
PLS-DA	7	142	0.736	0.841	0.645	25.7	< 0.05
OPLS-DA (Plant/thyme)	1+5+0	122	0.725	0.949	0.907	67.2	< 0.05
OPLS-DA (Plant/Blossom)	1+4+0	106	0.615	0.788	0.471	5.73	< 0.05
OPLS-DA (Thyme/Blossom)	1+4+0	56	0.718	0.987	0.915	42.4	< 0.05

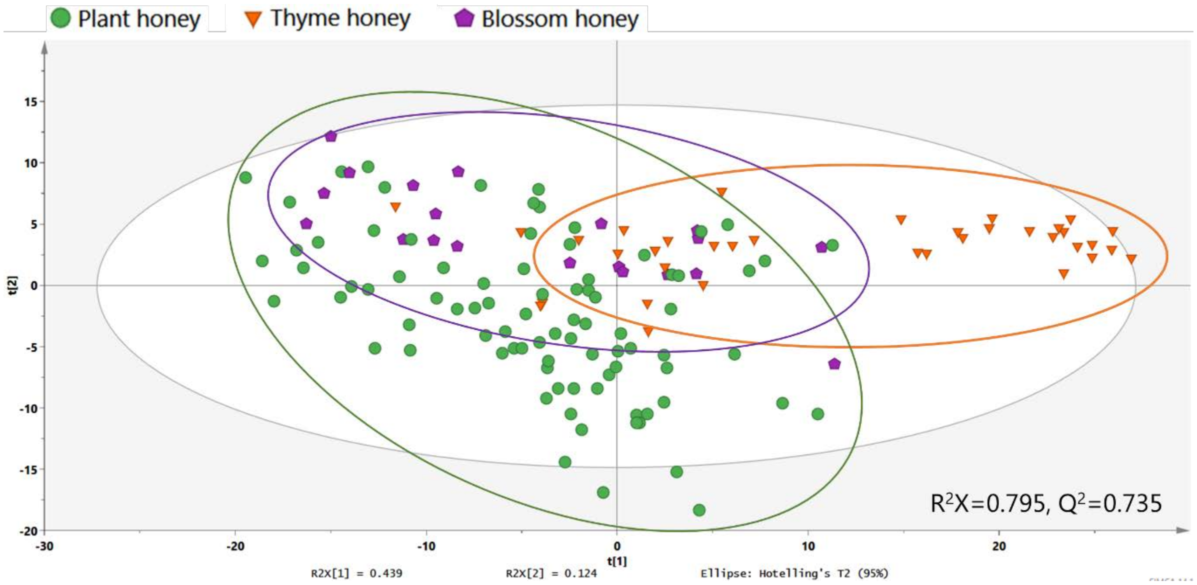

Figure S4. PCA scores scatter plot of the dataset showing the clustering based on botanical origin. Heather, chestnut and spring honeys were excluded due to their limited sample size.

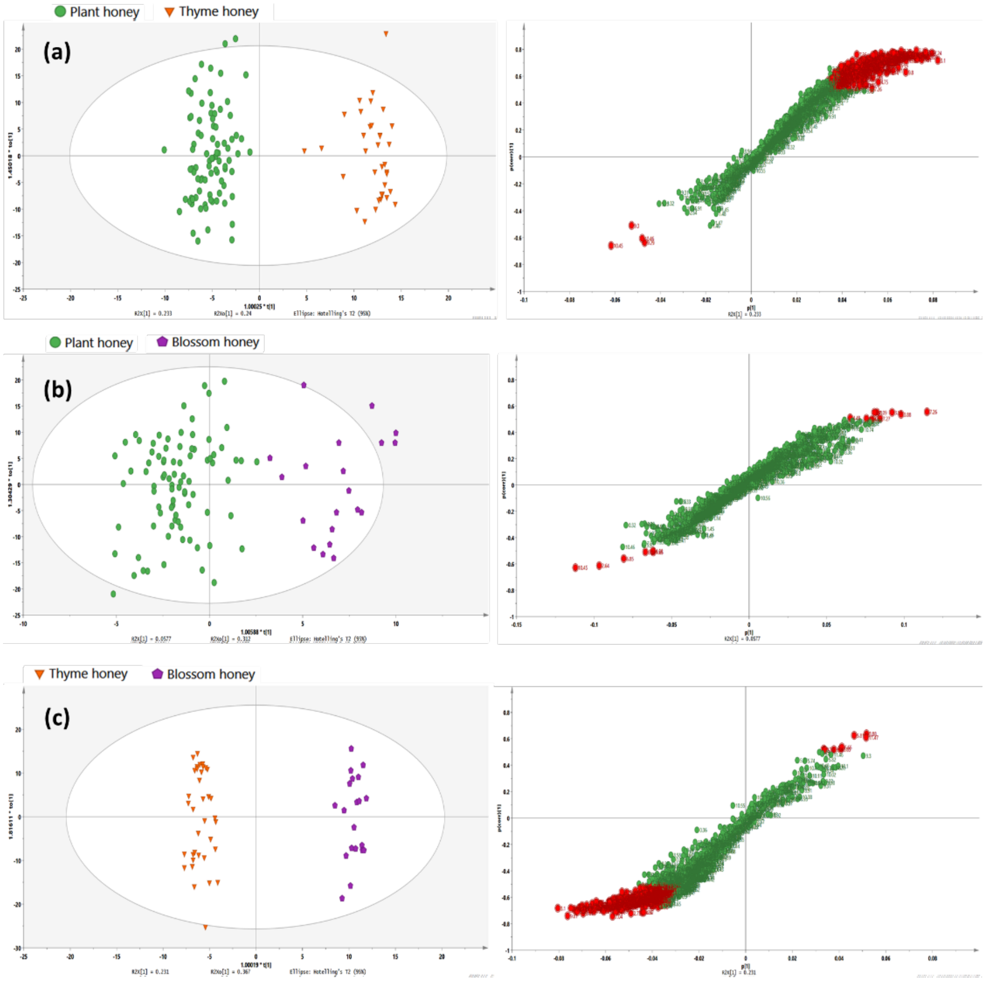

Figure S5. OPLS-DA scores scatter plots based on botanical origin with their respective S-plots. (**a**) Plant vs. Thyme, (**b**) Plant vs. Blossom, (**c**) Thyme vs. Blossom.

Table S3. List of models based on geographical origin. A, number of components; N, number of observations; cumulative R^2X, R^2Y, Q^2 values, F statistic and p-values determined from CV-ANOVA.

Model	A	N	R^2X	R^2Y	Q^2	F	p-value
PCA	13	138	0.820		0.741		
PLS-DA	11	138	0.789	0.902	0.807	19.4	< 0.05
OPLS-DA (Ikaria/Lesvos)	1+4+0	54	0.719	0.975	0.916	39.4	< 0.05
OPLS-DA (Ikaria/Lemnos)	1+3+0	22	0.817	0.994	0.970	46.8	< 0.05
OPLS-DA (Ikaria/Samos)	1+3+0	46	0.711	0.979	0.933	57.6	< 0.05
OPLS-DA (Ikaria/Fournoi)	1+2+0	28	0.814	0.984	0.964	85.4	< 0.05
OPLS-DA (Ikaria/Chios)	1+3+0	42	0.743	0.974	0.934	51.6	< 0.05
OPLS-DA (Lesvos/Lemnos)	1+4+0	52	0.621	0.986	0.918	38.1	< 0.05
OPLS-DA (Lesvos/Samos)	1+1+0	75	0.494	0.932	0.919	198.2	< 0.05
OPLS-DA (Lesvos/Fournoi)	1+2+0	58	0.672	0.979	0.956	181.9	< 0.05
OPLS-DA (Lesvos/Chios)	1+2+0	72	0.558	0.913	0.873	71.70	< 0.05
OPLS-DA (Lemnos/Samos)	1+1+0	43	0.479	0.967	0.954	194.1	< 0.05
OPLS-DA (Lemnos/Fournoi)	1+2+0	26	0.777	0.992	0.972	94.8	< 0.05
OPLS-DA (Lemnos/Chios)	1+3+0	40	0.556	0.993	0.959	79.3	< 0.05

OPLS-DA (Samos/Fournoi)	1+2+0	49	0.744	0.992	0.982	331.7	< 0.05
OPLS-DA (Samos/Chios)	1+3+0	64	0.589	0.967	0.924	69.7	< 0.05
OPLS-DA (Fournoi/Chios)	1+2+0	46	0.729	0.977	0.961	133.7	< 0.05

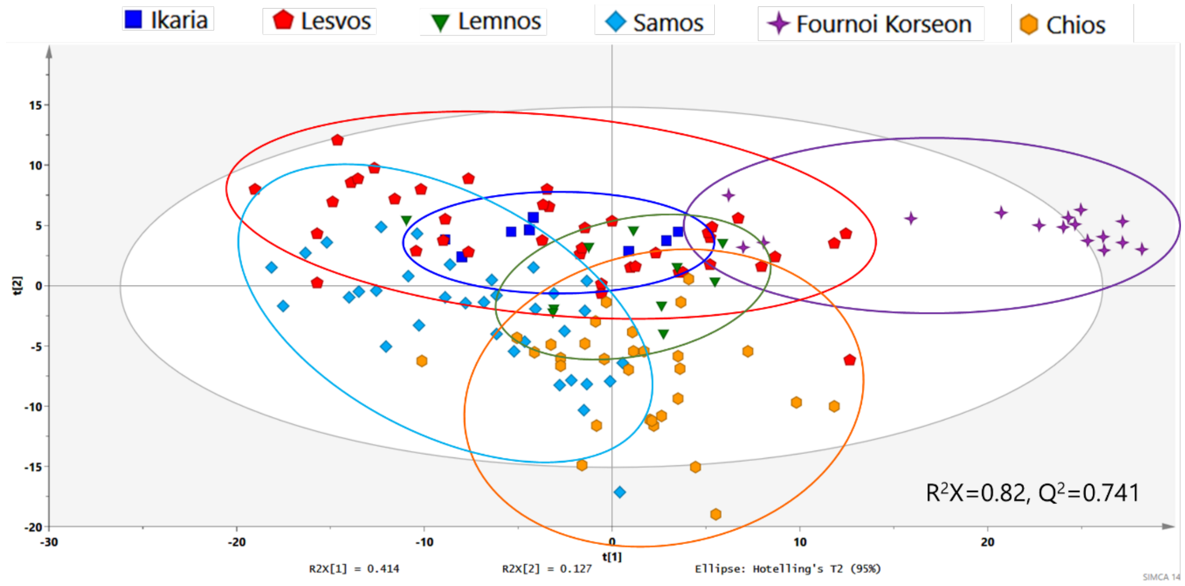

Figure S6. PCA scores scatter plot of the dataset showing the clustering based on geographical origin. Psara and Agios Efstratios honeys were excluded due to their limited sample size.

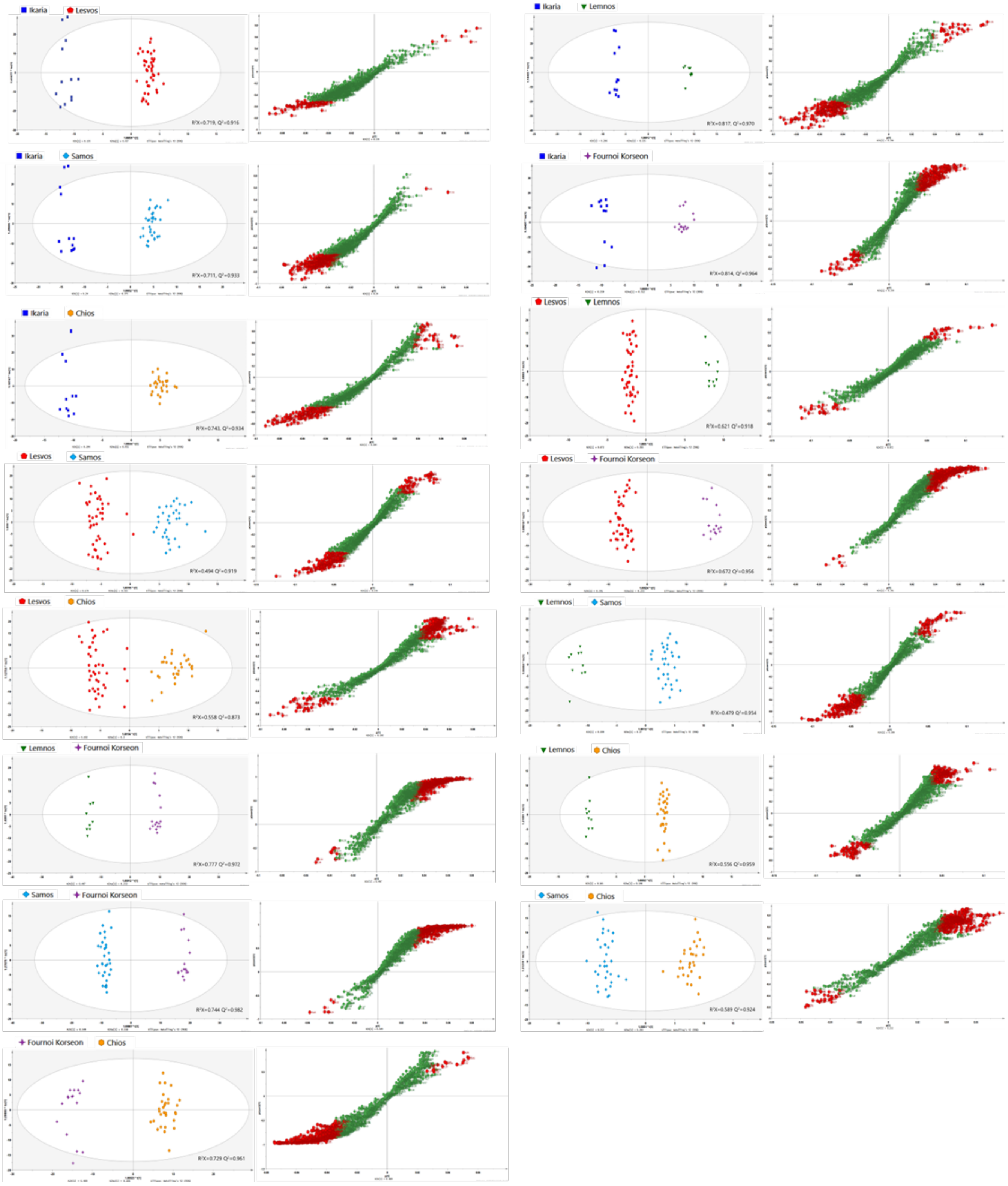

Figure S7. OPLS-DA scores scatter plots based on geographical origin with their respective S-plots.

Ultra-high performance liquid chromatography coupled to a hybrid resolution Mass spectrometer (UHPLC-HRMS & MS/MS)

Quality control of honey samples was carried out using a Velos Pro Ion Trap-Orbitrap Elite Hybrid Mass Spectrometer system (Thermo Scientific; Bremen, Germany), with a heated electrospray ion source (HESI) hyphenated to a Waters Acquity UPLC (Waters Corporation, Milford, MA, USA) system. The chromatographic column used was a Waters Acquity UPLC C18 (2.1x150m, 1.7um) adjusted at 40°C. The total running time of the method was 30 min, and the injection volume was 10 uL. All samples were prepared at a concentration of 500 ug/mL. The elution method is described in the table below.

Time (min)	Solvent A (H_2O+0.1%FA)	Solvent B (ACN)	Flow (mL/min)
0	95	5	0.3
1	95	5	0.3
3	80	20	0.3
20	40	60	0.3
23	0	100	0.3
27	0	100	0.3
28	95	5	0.3
30	95	5	0.3

Data were obtained in negative and positive ionization mode, at a scan range of *m/z* 113-1000. HESI temperature, capillary interface and source heater temperature was set at 350 °C, while source electrospray voltage was 3.5 kV, S-lens RF level 45% and 60% for negative and positive polarity, respectively. Nitrogen was used as sheath and auxiliary gas with flows set at 45 and 15 arbitrary units, respectively. A data-depended acquisition (DDA) method was set up involving two scan events: a full MS scan followed by HRMS/MS acquisition. Full-scan spectra were acquired at high resolution of 60,000 full width half maximum (FWHM) whereas HRMS/MS acquisition were acquired in mass resolution 17,500 FWHM with normalized collision energy (NCE) of 35%. The entire system control and data processing were performed with Xcalibur 2.2 (Thermo Electron, San Jose, CA, USA) software.

Table S4. Identified compounds using HRMS and HRMS/MS in negative ionization mode.

Compounds	Molecular Formula	Experimental [M-H]$^-$ *m/z*	Theoretical [M-H]$^-$ *m/z*	*Δ*m (ppm)	RDBeq	MSMS Fragments (*Relative intensity %*)
4-MBZA	$C_8H_8O_3$	151.0399	151.0401	-0.794	5.5	n.d
Unedone	$C_{13}H_{20}O_4$	239.1287	239.1287	-0.794	4.5	n.d
Dehydrovomifoliol	$C_{13}H_{18}O_3$	-	-	-	-	-
BZA	$C_7H_6O_2$	121.0294	121.0295	-0.756	5.5	n.d
ABA	$C_{15}H_{20}O_4$	263.1289	263.1289	0.091	6.5	219 (100), 201 (37), 152 (12).
1-(4-methoxyphenyl)-ethane-1,2-diol	$C_9H_{12}O_3$	167.0713	167.0714	-0.287	4.5	n.d
MSYR	$C_{10}H_{12}O_5$	163.0763	163.0765	-0.694	5.5	n.d
3-hydroxy-4-phenyl-2- butanone	$C_{10}H_{12}O_2$	163.0764	163.0765	-0.039	5.5	n.d
5-HMF	$C_6H_6O_3$	125.0244	125.0244	-0.270	4.5	n.d

Table S5. Identified compounds using HRMS and HRMS/MS in positive ionization mode.

Compounds	Molecular Formula	Experimental [M-H]- *m/z*	Theoretical [M-H]- *m/z*	*Δ*m (ppm)	RDBeq	MSMS Fragments (*Relative intensity %*)
4-MBZA	$C_8H_8O_3$	153.0547	153.0546	0.337	4.5	135 (100)
Unedone	$C_{13}H_{20}O_4$	241.1432	241.1434	0.772	3.5	197 (100), 223 (80), 205 (42)
Dehydrovomifoliol	$C_{13}H_{18}O_3$	223.1329	223.1329	0.012	4.5	205 (100), 165 (99), 187 (42)
BZA	$C_7H_6O_2$	123.0441	123.0441	0.032	4.5	n.d
ABA	$C_{15}H_{20}O_4$	265.1433	265.1434	0.357	5.5	209 (100), 247 (73), 229 (33)
1-(4-methoxyphenyl)-ethane-1,2-diol	$C_9H_{12}O_3$	-	-	-	-	-
MSYR	$C_{10}H_{12}O_5$	213.0755	213.0757	1.386	4.5	181 (100)
3-hydroxy-4-phenyl-2- butanone	$C_{10}H_{12}O_2$	165.0911	165.0910	0.445	4.5	137 (100)
5-HMF	$C_6H_6O_3$	127.0391	127.0390	0.904	3.5	n.d

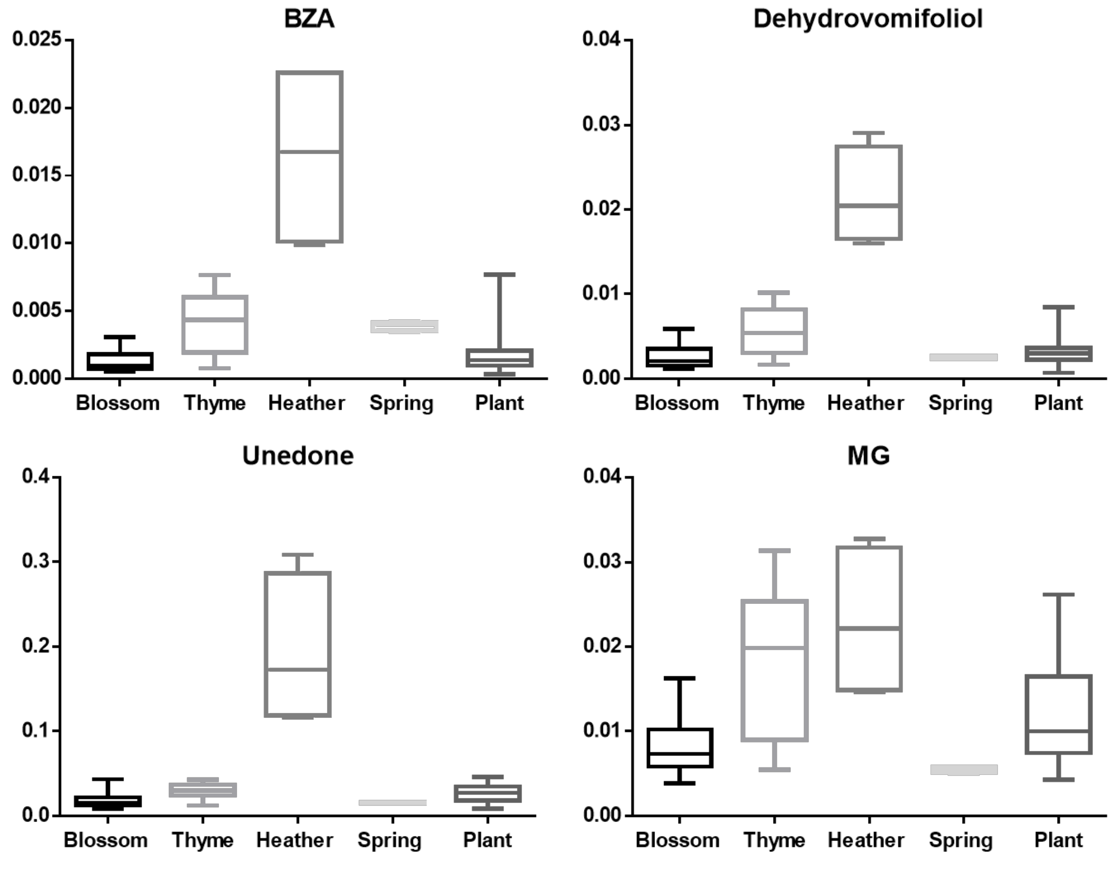

Figure S8. Box plots of statistically significant biomarkers based on their botanical origin are presented. All p values can be found in Table S6.

Table S6. Box Plot´s p values comparing all the possible groups based on Botanical origin. Statistically significant, p value ≤ 0.05.

Groups - Botanical Origin	Significant	Adjusted P Value
**5-HMF**		
Blossom vs. Thyme	Yes	0.0029
Blossom vs. Heather	No	0.6079
Blossom vs. Spring	Yes	0.0233
Blossom vs. Plant	Yes	0.0078
Thyme vs. Heather	Yes	0.0068
Thyme vs. Spring	Yes	<0.0001
Thyme vs. Plant	Yes	<0.0001
Heather vs. Spring	No	0.7259
Heather vs. Plant	No	>0.9999
Spring vs. Plant	No	0.4959
**MSYR**		
Blossom vs. Thyme	Yes	0.0002
Blossom vs. Heather	No	0.1330
Blossom vs. Spring	Yes	0.0452
Blossom vs. Plant	Yes	0.0007
Thyme vs. Heather	No	>0.9999
Thyme vs. Spring	No	0.9770
Thyme vs. Plant	No	0.8201
Heather vs. Spring	No	0.9970
Heather vs. Plant	No	0.9822
Spring vs. Plant	No	0.8459
**MG Derivative**		
Blossom vs. Thyme	Yes	<0.0001
Blossom vs. Heather	Yes	0.005
Blossom vs. Spring	No	0.9156
Blossom vs. Plant	No	0.1631
Thyme vs. Heather	No	0.6824
Thyme vs. Spring	Yes	0.0013
Thyme vs. Plant	Yes	<0.0001
Heather vs. Spring	Yes	0.0014
Heather vs. Plant	Yes	0.0081
Spring vs. Plant	No	0.2691
**3-hydroxy-4-phenyl-2-butanone**		
Blossom vs. Thyme	Yes	<0.0001
Blossom vs. Heather	No	0.7343
Blossom vs. Spring	No	>0.9999
Blossom vs. Plant	No	>0.9999
Thyme vs. Heather	No	0.1235
Thyme vs. Spring	Yes	0.0022
Thyme vs. Plant	Yes	<0.0001
Heather vs. Spring	No	0.8411
Heather vs. Plant	No	0.6709

Groups - Botanical Origin	Significant	Adjusted P Value
Spring vs. Plant	No	>0.999
**Unedone**		
Blossom vs. Thyme	No	0.1307
Blossom vs. Heather	Yes	<0.0001
Blossom vs. Spring	No	0.9872
Blossom vs. Plant	No	0.2812
Thyme vs. Heather	Yes	<0.0001
Thyme vs. Spring	No	0.3940
Thyme vs. Plant	No	0.9149
Heather vs. Spring	Yes	<0.0001
Heather vs. Plant	Yes	<0.0001
Spring vs. Plant	No	0.5655
**ABA**		
Blossom vs. Thyme	No	0.9992
Blossom vs. Heather	Yes	<0.0001
Blossom vs. Spring	No	0.9757
Blossom vs. Plant	No	0.9582
Thyme vs. Heather	Yes	<0.0001
Thyme vs. Spring	No	0.9883
Thyme vs. Plant	No	0.9853
Heather vs. Spring	Yes	<0.0001
Heather vs. Plant	Yes	<0.0001
Spring vs. Plant	No	0.9982
**Dehydrovomifoliol**		
Blossom vs. Thyme	Yes	<0.0001
Blossom vs. Heather	Yes	<0.0001
Blossom vs. Spring	No	>0.9999
Blossom vs. Plant	No	0.8480
Thyme vs. Heather	Yes	<0.0001
Thyme vs. Spring	No	0.0297
Thyme vs. Plant	Yes	<0.0001
Heather vs. Spring	Yes	<0.0001
Heather vs. Plant	Yes	<0.0001
Spring vs. Plant	No	0.9769
**4-MBA**		
Blossom vs. Thyme	No	0.9047
Blossom vs. Heather	Yes	<0.0001
Blossom vs. Spring	No	>0.9999
Blossom vs. Plant	No	0.9984
Thyme vs. Heather	Yes	<0.0001
Thyme vs. Spring	No	0.9975
Thyme vs. Plant	No	0.9074
Heather vs. Spring	Yes	<0.0001
Heather vs. Plant	Yes	<0.0001
Spring vs. Plant	No	>0.9999

Groups - Botanical Origin	Significant	Adjusted P Value
**BZA**		
Blossom vs. Thyme	Yes	<0.0001
Blossom vs. Heather	Yes	<0.0001
Blossom vs. Spring	No	0.0680
Blossom vs. Plant	No	0.9067
Thyme vs. Heather	Yes	<0.0001
Thyme vs. Spring	No	>0.9999
Thyme vs. Plant	Yes	<0.0001
Heather vs. Spring	Yes	<0.0001
Heather vs. Plant	Yes	<0.0001
Spring vs. Plant	No	0.1193
**1-(4-methoxyphenyl)-ethane-1,2-diol**		
Blossom vs. Thyme	No	0.1772
Blossom vs. Heather	Yes	<0.0001
Blossom vs. Spring	No	0.9972
Blossom vs. Plant	No	0.9653
Thyme vs. Heather	Yes	<0.0001
Thyme vs. Spring	No	0.9224
Thyme vs. Plant	No	0.1501
Heather vs. Spring	Yes	<0.0001
Heather vs. Plant	Yes	<0.0001
Spring vs. Plant	No	>0.9999

Table S7. Box Plot´s p values comparing thyme honey samples with different geographical origin. Statistically significant, p value ≤ 0.05.

Groups (Geographical Origin)	Significant	Adjusted p Value
**5-HMF**		
Ikaria vs. Lemnos	Yes	0.0069
Ikaria vs. Fournoi	Yes	0.0164
Ikaria vs. Psara	No	0.1602
Lemnos vs. Fournoi	No	0.8719
Lemnos vs. Psara	No	0.5393
Fournoi vs. Psara	No	0.8465
**MSYR**		
Ikaria vs. Lemnos	No	0.7381
Ikaria vs. Fournoi	Yes	0.0468
Ikaria vs. Psara	Yes	< 0.0001
Lemnos vs. Fournoi	No	0.1195
Lemnos vs. Psara	Yes	< 0.0001
Fournoi vs. Psara	Yes	0.0013
**MG Derivative**		
Ikaria vs. Lemnos	No	0.4990
Ikaria vs. Fournoi	Yes	0.0007
Ikaria vs. Psara	Yes	0.0049

Groups (Geographical Origin)	Significant	Adjusted p Value
Lemnos vs. Fournoi	Yes	0.0020
Lemnos vs. Psara	Yes	0.0295
Fournoi vs. Psara	No	0.9978
**3-hydroxy-4-phenyl-2-butanone**		
Ikaria vs. Lemnos	No	0.9967
Ikaria vs. Fournoi	Yes	< 0.0001
Ikaria vs. Psara	No	0.2563
Lemnos vs. Fournoi	Yes	< 0.0001
Lemnos vs. Psara	No	0.0650
Fournoi vs. Psara	Yes	0.0026

Table S8. Box Plot´s p values comparing plant honey samples with different geographical origin. Statistically significant, p value ≤ 0.05.

Groups (Geographical Origin)	Significant	Adjusted P Value
**5-HMF**		
Lesvos vs. Samos	Yes	<0.0001
Lesvos vs. Chios	No	0.1233
Samos vs. Chios	Yes	<0.0001
**MSYR**		
Lesvos vs. Samos	Yes	<0.0001
Lesvos vs. Chios	Yes	<0.0001
Samos vs. Chios	No	0.7717
**MG Derivative**		
Lesvos vs. Samos	Yes	0.0340
Lesvos vs. Chios	No	0.8958
Samos vs. Chios	Yes	0.0032
**3-hydroxy-4-phenyl-2-butanone**		
Lesvos vs. Samos	Yes	0.0003
Lesvos vs. Chios	No	0.2287
Samos vs. Chios	Yes	0.0231
